# Electrocatalytic Reduction of Dinitrogen to Ammonia with Water as Proton and Electron Donor Catalyzed by a Combination of a Tri-ironoxotungstate and an Alkali Metal Cation

**DOI:** 10.1021/jacs.3c06167

**Published:** 2023-08-29

**Authors:** Avra Tzaguy, Albert Masip-Sánchez, Liat Avram, Albert Solé-Daura, Xavier López, Josep M. Poblet, Ronny Neumann

**Affiliations:** † Department of Molecular Chemistry and Materials Science, Weizmann Institute of Science, Rehovot, Israel 76100; ‡ Department de Química Física i Inorgànica, Universitat Rovira i Virgili, Tarragona 43007, Spain; § Department of Chemical Research Support, Weizmann Institute of Science, Rehovot, Israel 76100

## Abstract

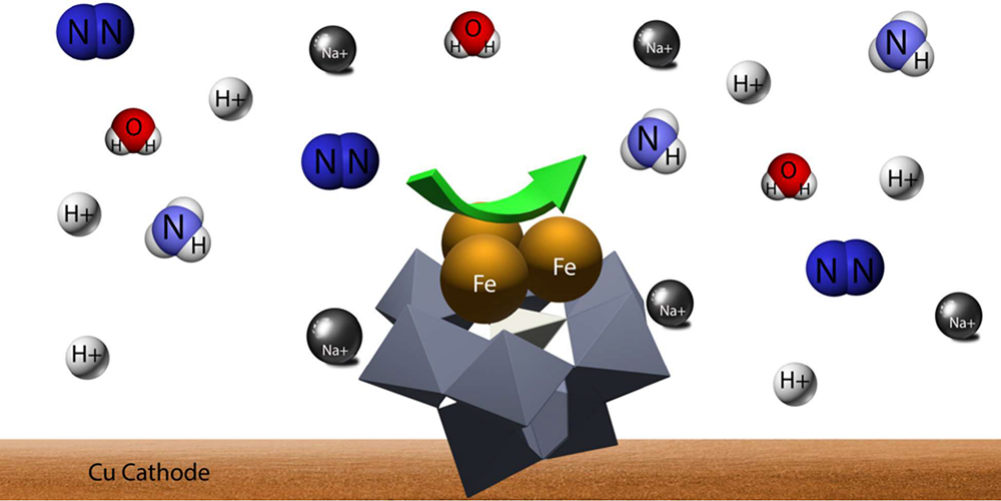

The electrification of ammonia synthesis is a key target for its decentralization and lowering impact on atmospheric CO_2_ concentrations. The lithium metal electrochemical reduction of nitrogen to ammonia using alcohols as proton/electron donors is an important advance, but requires rather negative potentials, and anhydrous conditions. Organometallic electrocatalysts using redox mediators have also been reported. Water as a proton and electron donor has not been demonstrated in these reactions. Here a N_2_ to NH_3_ electrocatalytic reduction using an inorganic molecular catalyst, a tri-iron substituted polyoxotungstate, {SiFe_3_W_9_}, is presented. The catalyst requires the presence of Li^+^ or Na^+^ cations as promoters through their binding to {SiFe_3_W_9_}. Experimental NMR, CV and UV–vis measurements, and MD simulations and DFT calculations show that the alkali metal cation enables the decrease of the redox potential of {SiFe_3_W_9_} allowing the activation of N_2_. Controlled potential electrolysis with highly purified ^14^N_2_ and ^15^N_2_ ruled out formation of NH_3_ from contaminants. Importantly, using Na^+^ cations and polyethylene glycol as solvent, the anodic oxidation of water can be used as a proton and electron donor for the formation of NH_3_. In an undivided cell electrolyzer under 1 bar N_2_, rates of NH_3_ formation of 1.15 nmol sec^–1^ cm^–2^, faradaic efficiencies of ∼25%, 5.1 equiv of NH_3_ per equivalent of {SiFe_3_W_9_} in 10 h, and a TOF of 64 s^–1^ were obtained. The future development of suitable high surface area cathodes and well solubilized N_2_ and the use of H_2_O as the reducing agent are important keys to the future deployment of an electrocatalytic ammonia synthesis.

## Introduction

Humankind is dependent on the manufacture of ammonia and its derivatives as fertilizers for food production.^1,2^ The highly optimized heterogeneous catalytic process, N_2_ + 3H_2_ → 2 NH_3_, is only feasible at high temperatures and pressures using very high purity N_2_ and H_2_.^3^ Hydrogen is produced via a high energy consuming steam reforming reaction from natural gas, and it is estimated that ∼1% of the world’s energy consumption and 1.5% of global CO_2_ emissions are directly from the Haber–Bosch (H–B) process.^2,3^ Thus, NH_3_ is produced at locations where natural gas is plentiful but not necessarily where the end users are located. Future availability of renewable electricity suggests two options to replace the traditional H–B process. The hybrid-H–B approach uses H_2_ from water electrolysis for H–B NH_3_ synthesis, which obviates the use of natural gas and reduces the overall carbon footprint, leading to decarbonization of the process. Another option is electrocatalytic NH_3_ synthesis that proceeds via an electrochemical Nitrogen Reduction Reaction (e-N2RR) obtaining the needed protons and electrons from water oxidation. In contrast to the hybrid-H–B, e-N2RR is viable for the cleavage of the high energy N≡N bond to NH_3_ at ambient conditions, and a catalytic reaction can be feasible under much more benign conditions. In fact, the nitrogenase enzyme complex reduces N_2_ to NH_3_, albeit quite inefficiently using 16 equiv of ATP (Adenosine triphosphate) per N_2_ molecule.^4^ An economic analysis of hybrid H–B and electrocatalytic NH_3_ approaches shows that the former would be economically feasible at large production scales, but electrocatalytic NH_3_ would outperform at small production scales (∼0.03 ton NH_3_/day).^5^ Climate benefits of e-NH_3_ include a reduced carbon footprint associated with reduced maritime and overland transportation, reduced storage needs, enhanced ability to follow the intermittent electrical power input, and use of nitrogen with reduced purity. These factors make decentralized ammonia production an attractive long-term option.^6^ On-site, on-demand NH_3_ production can improve decarbonization of agricultural and shipping sectors and be more resistant against political-economic risks.^7^

Nitrogenase enzymes convert N_2_ to NH_3_ under ambient reaction conditions. The most studied enzyme has an active site with a hepta-iron–molybdenum, FeMo, cofactor. The accepted overall reaction equation is eq 1. This eight-electron process is coupled to ATP hydrolysis and accompanied by the formation of 1 mol of H_2_ per mole of N_2_ reduced.
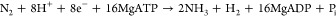
1

Nitrogenase catalyzed N_2_ reduction to NH_3_ (N2RR) was shown to have a turnover frequency (TOF) of about 2 NH_3_ s^–1^ and a high turnover number (TON > 10^6^). Numerous studies were consolidated into the consensus Lowe and Thorneley (LT) kinetic model that defines how electrons and protons are accumulated at the active FeMo-cofactor site.^8^ Identification of the reaction intermediates in the enzyme is very complicated, leading to exploration of surrogate coordination compounds as (very) simplified models. Studies of catalytic N_2_-to-NH_3_ conversion by model complexes enable analysis of hypotheses concerning the mechanism of N_2_-fixation and define catalyst design principles for the multielectron reductive transformations.^9,10^ The high stability of the N≡N triple bond requires catalysts and high-energy reagents/conditions to form NH_3_ through associative and dissociative reaction mechanisms.^11,12^ Iron model systems that catalyze N_2_-to-NH_3_ conversion have been studied based on the hypothesis that one or more Fe centers in the FeMo-nitrogenase serve as the site of N_2_ binding and activation during the N≡N bond-breaking and N–H bond formation steps.^4,13−15^ Synthetic molecular Fe catalysts that mediate N_2_-to-NH_3_ conversion typically operate with high driving forces, using very strong acids (p*K*_a_ ca. 0) and reductants (*E*° ≤ −3.0 V vs Fc^+/0^).^16−21^ Contrarily, Mo-based catalysts have been shown to facilitate N_2_-to-NH_3_ conversion with lower driving forces.^22−24^ Recently, milder reductants (Cp*_2_Co, *E*° ≤ −1.96 V vs Fc^+/0^) and acids such as diphenylanalinium triflate as the proton donor were also shown to be efficient in homogeneous N2RR using an iron catalyst.^25^

Despite the advances toward understanding both N_2_ activation and NH_3_ formation, electrocatalytic reduction to NH_3_ and (electro) catalyst development is in its infancy.^26−28^ Recent critiques of reported electrocatalytic N_2_RR reactions mostly carried out in water with various electrode materials concluded that most reported research could not be verified for a combination of reasons related to too low yields for reliable analysis, NO_*x*_ impurities in the N_2_ gas used as reagent, contamination with ambient ammonia, and other deficiencies.^29−31^ Based on the known reactivity of Li metal with N_2_, electrochemical NH_3_ synthesis is possible via a set of three reactions carried out separately, eqs 2–4.^32^ Based on research from the 1990s,^33,34^ a cascade of such reactions can be carried out in a single electrochemical cell. Typically, ethanol has been used as a presumed source of protons/electrons in THF.^29,35,36^ However, very recently it has been reported that alcohols are in fact proton carriers suggesting that THF is the major proton/electron source.^37^ The use of a phosphonium cation as a proton carrier^38^ has also been reported. Reactions in the presence of other alcohols,^39,40^ and H_2_ have also been reported.^41,42^
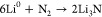
2
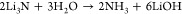
3
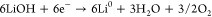
4

Catalytic cycles for the transformation of N_2_ to NH_3_ have been reported using a variety of coordination compounds in homogeneous reaction media as noted above, but only rarely have such molecular catalytic reactions been electrified.^43^ Tandem electrocatalysis has been demonstrated for reactions in solution with a cobaltocene-based PCET mediator using an Fe(tris(*o*-diisopropylphosphinophenyl)-borane catalyst and diphenylanalinium triflate as the proton donor,^44^ and using a bis(diphenylphosphinoethane)tungsten catalyst and toluene sulfonic acid as the proton donor.^45^ Interestingly, a molybdenum(III) pincer compound electrocatalytically reduced N_2_ to NH_3_ using collidinium triflate as the preferred proton source, wherein the PCET mediator showed no synergetic effect.^46^

**Figure 1:**
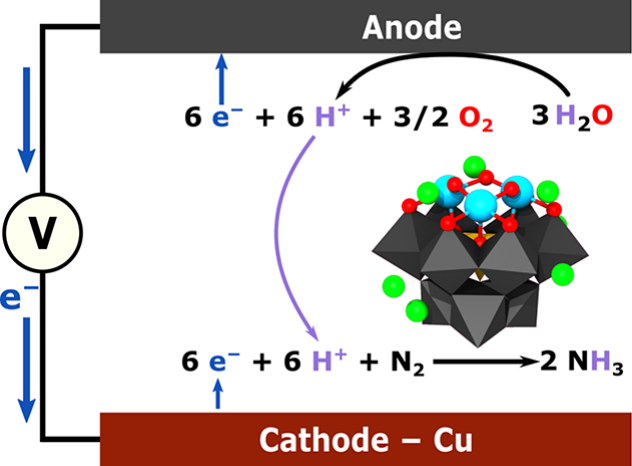
Schematic presentation of sustainable electrosynthesis of NH_3_ from H_2_O and N_2_ using {SiFe^III^_3_W_9_} as electrocatalyst and an alkali cation as promotor. N_2_ + 3 H_2_O → 2 NH_3_ + 3/2 O_2_. Colors: Fe – cyan; Si – yellow; W – gray; O – red; Na/Li – green.

Polyoxometalates are attractive inorganic catalysts because they are easy to synthesize, their intrinsic properties may be easily modified, and they can be used with excellent efficiency in transformations involving electron transfer.^47^ Furthermore, many of these polyoxometalates display reversible redox processes that are sensitive to the presence of protons. Although they are weak bases and nucleophiles, polyoxometalates can promote the formation of hydrogen-bond networks in the vicinity of a substrate coordination site to favor proton coupled electron transfer. Inclusion of redox transition metals in polyoxometalates increases the reactivity of the polyanion, which has surfaces that are populated with weakly basic oxygen atoms. Adapting these properties of polyoxometalates and using a rational design approach based on the multi-iron active site of the nitrogenase enzyme, we have used a tri-iron containing polyoxometalate, [SiW_9_Fe^III^_3_(H_2_O)_3_O_37_]^7–^, {SiFe^III^_3_W_9_},^48^ to show that N_2_ binds to and is activated by {SiFe^III^_3_W_9_} in the presence of Li^+^. Further, selective e-N2RR of N_2_ to NH_3_ was demonstrated in anhydrous solvents using ethanol as the proton/electron donor and then in polyethylene glycol (PEG-400) using Na^+^ as the promoter and with up to 1 vol % water as the proton/electron donor at ∼ –1.3 V vs SHE, Figure 1.

## Results and Discussion

Bioinspired by the hepta-iron site of nitrogenase, the tri-iron substituted polyoxometalate with a tetrabutyl ammonium cation (TBA), TBA_7_[SiFe_3_W_9_O_37_]·3TBA, TBA{SiFe_3_W_9_} was first studied for its reactivity toward the binding and activation of N_2_.

**Figure 2:**
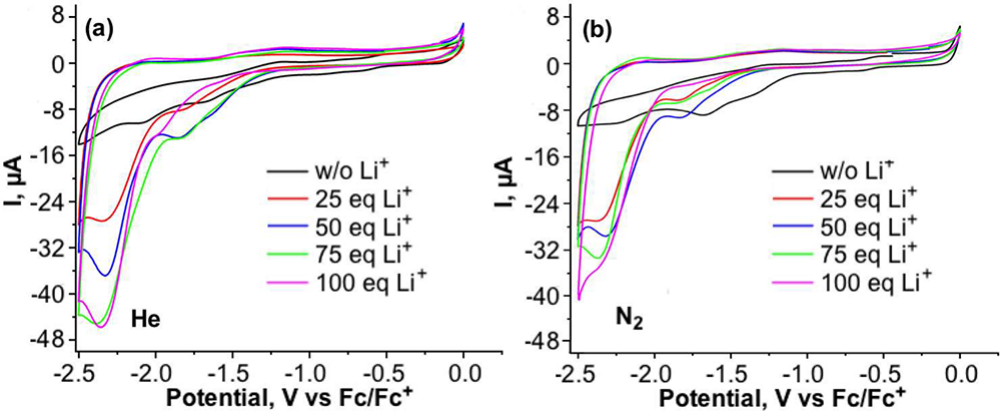
Cyclic Voltammetry of TBA{SiFe_3_W_9_} and Effect of Li^+^. The cyclic voltammetry measurements, 100 mV/s, were carried out in 10 mL of dry THF containing 0.1 M TBAPF_6_ and 0.5 mM TBA{SiFe_3_W_9_}with various amounts of LiClO_4_ under 1 bar He (a) or N_2_ (b) with a glassy carbon disc working electrode, a platinum wire counter electrode, and a Fc/Fc^+^ reference electrode.

**Figure 3:**
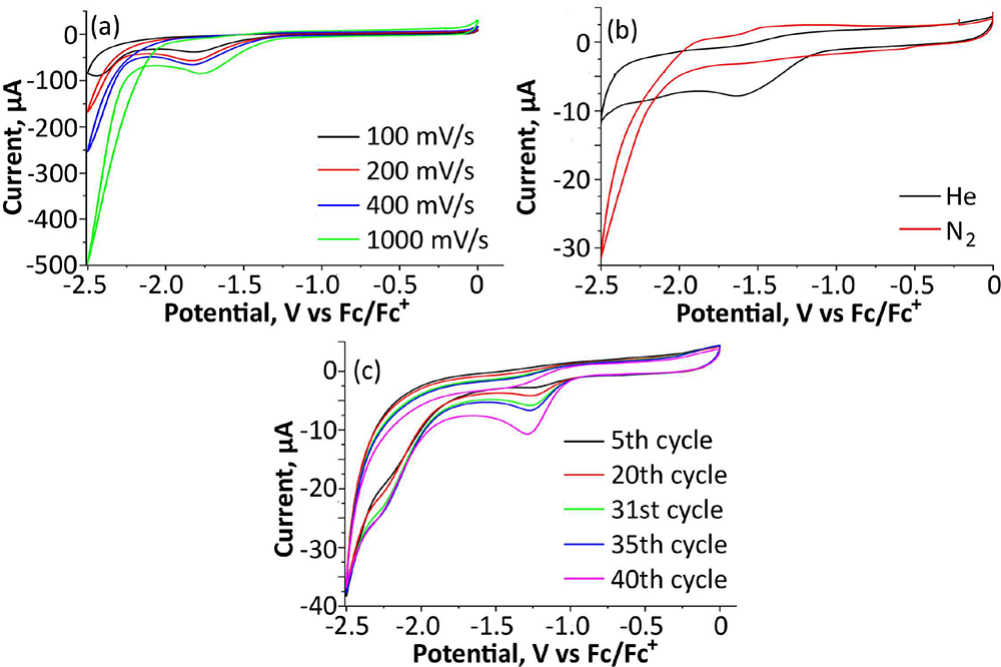
Cyclic Voltammetry of TBA{SiFe_3_W_9_} under N_2_ in the presence of ethanol: (a) as a function of scan rates; (b) one scan under 1 bar He and then one scan under 1 bar N_2_; (c) as a function of the number of cycles. The cyclic voltammetry measurements were carried out in 10 mL of 99:1 dry THF:ethanol containing 100 mM TBAPF_6_ and 0.5 mM TBA{SiFe_3_W_9_}, 50 mM LiClO_4_ under 1 bar N_2_ with a glassy carbon disc working electrode, a platinum wire counter electrode, and a Fc/Fc^+^ reference electrode; scan rates for (b) and (c) = 100 mV/s.

Cyclic voltammetry (CV) experiments showed that although very similar voltammograms were obtained under He as a control and N_2_, the addition of Li^+^ as LiClO_4_ to a THF solution of 0.5 mM TBA{SiFe_3_W_9_}and 0.1 M TBAPF_6_ as electrolyte led to the appearance of reduction peaks, whose intensity increased as a function of the amount of Li^+^ added at around −1.80 and −2.30 V versus Fc/Fc^+^, Figure 2. The interpretation of these initial measurements is that the presence of Li^+^ as a cation considerably influences the redox potential of TBA{SiFe_3_W_9_} as will be further discussed below. In the absence of a proton or electron donor such as ethanol, electrocatalytic reduction of N_2_ is unlikely. Thus, a further cyclic voltammetry measurement first under 1 bar of He and then under 1 bar of N_2_ in the presence of 1% ethanol as a proton/electron donor showed an increase in the peak current at −2.5 V versus Fc/Fc^+^, Figure 3a, compared to the current in the absence of ethanol, Figure 2b. An onset potential of −1.85 V of Fc/Fc^+^ is associated with N_2_ reduction. Application of the Randles–Sevcik equation, Figure S1, shows a linear correlation between the absolute value of the peak current and the square root of the scan rate, demonstrating reversible electron transfer. Although cyclic voltammetry measurements at 1 bar He under the conditions reported in Figures 2 and 3 are only possible for a few scans, since TBA{SiFe_3_W_9_}, Li^+^ visibly degrades with formation of precipitates from solution, we carried out an experiment where one cyclic voltammetry scan was first measured under 1 bar He, followed by one scan under 1 bar N_2_, Figure 3b. Also, here a catalytic peak was observed in the presence of N_2_ with an onset potential of −1.85 V Fc/Fc^+^.

In addition, repeated CV cycles were measured in the presence of 1% ethanol at a scan rate of 100 mV/s (Figure 3c). It can be observed that the peak current, −2.5 V versus Fc/Fc^+^, associated with N_2_ reduction, remains unchanged over 40 cycles. On the other hand, two additional peaks appeared at −1.3 and −2.2 V versus Fc/Fc^+^ over time. The peak at −1.3 V is associated with the formation of acetaldehyde, the two-electron oxidation product of ethanol, as shown in a separate CV measurement, Figure S2. The assignment of the peak at −2.2 V is uncertain but is preliminarily hypothesized to be related to the formation of a reaction intermediate between TBA{SiFe_3_W_9_}, Li^+^ and N_2_. Together all these cyclic voltammetry measurements are an excellent indication of the reaction between reduced TBA{SiFe_3_W_9_}, Li^+^ and N_2_.

**Figure 4:**
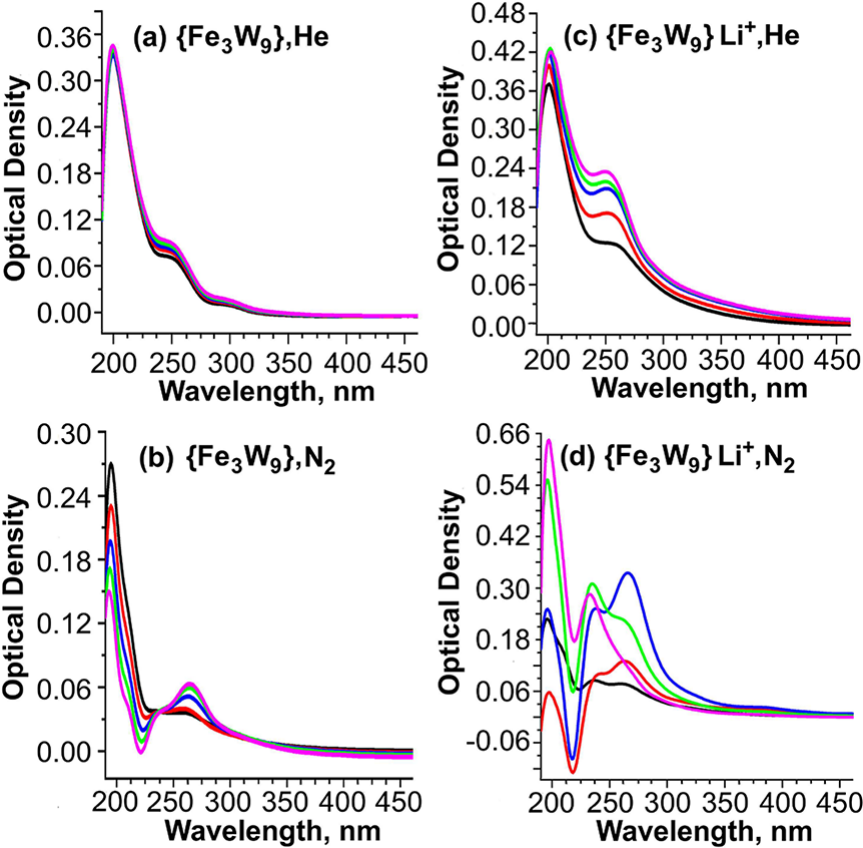
UV–vis spectra of {SiFe_3_W_9_} without Li^+^ (a,b) and with Li^+^ (c,d) under He (a,c) or N_2_ (b,d). The measurements were made using 4 μM TBA{SiFe_3_W_9_}, 0.01 M TBAPF_6_, and 200 μM LiClO_4_ (c, d) in THF. The reference solution was 0.01 M TBAPF_6_ in THF under 1 bar gas. In situ electrolysis was carried out in a 1 cm quartz cuvette using a Pt gauze working electrode, a Pt wire counter electrode, and a Ag wire as reference electrode at −2.1 V versus Ag (−2.01 V versus Fc/Fc^+^ by calibration, Figure S4). Black: before electrolysis; red: 1-electron per TBA{SiFe_3_W_9_}; blue: 2-electron per TBA{SiFe_3_W_9_}; green: 3-electron per TBA{SiFe_3_W_9_} and magenta: 4-electron per TBA{SiFe_3_W_9_}.

There was significant formation of NH_3_ from N_2_ at a potential of −2.0 V versus Fc/Fc^+^ (see below), which, as noted above, suggests an interaction of N_2_ with TBA{SiFe_3_W_9_} in the presence of Li^+^. UV–vis measurements related to the intense LMCT peaks of TBA{SiFe_3_W_9_} (log ε > 4.2) at −2.0 V versus Fc/Fc^+^ under controlled potential electrolysis (CPE) conditions, where TBA{SiFe_3_W_9_} is reduced, were carried out, Figure 4.

A control experiment under He and in the absence of Li^+^ shows a peak at 250 nm with a shoulder at 295 nm, which is essentially unchanged as the number of electrons added to the solution increases, Figure 4a. In a complementary experiment under N_2_, TBA{SiFe_3_W_9_} undergoes a transformation manifested by a new spectrum with a peak at 276 nm and a decrease in intensity at 222 nm accompanied by an isosbestic point at 233 nm, Figure 4b. Since there is no reduction of N_2_ to NH_3_ with TBA{SiFe_3_W_9_} only (see below), this spectrum could be associated with a yet unspecified interaction of N_2_ with TBA{SiFe_3_W_9_}. Another control experiment in the presence of TBA{SiFe_3_W_9_} and Li^+^ under reducing conditions and under He shows a spectrum very similar to that obtained with TBA{SiFe_3_W_9_} alone but with a somewhat higher intensity and the absence of the shoulder at 296 nm, Figure 4c. As will be discussed below, this change is likely due to the strong interaction between TBA{SiFe_3_W_9_} and Li^+^. Finally, the spectra obtained with TBA{SiFe_3_W_9_} and Li^+^ under N_2_ revealed continuous changes as the number of electrons introduced increases, as shown in Figure 4d. After a 1-electron reduction there is a decrease in intensity at 217 nm with stronger peaks at 235 and 267 nm (red). These latter peaks are intensified upon further 2-electron reduction (blue). A 3-electron reduction (green) leads to a positive change intensity at 217 and 230 nm and a negative change at 266 nm. Finally, 4-electron reduction (magenta) shows a dominant spectral feature at 233 nm. No further changes are observed in the spectra upon further reduction. It is not possible to assign the specific intermediates to the various spectra obtained, but a progression of formation of different species seems apparent. Opening the solution containing TBA{SiFe_3_W_9_}, Li^+^ and N_2_, Figure 4d, after 4-electron reduction to air, led to the appearance of the original pre-electrolysis spectrum with some residual 1-electron reduced species, Figure S3. Various spectroscopic methods attempted for further characterization of possible intermediates such as Mössbauer and 1D and 2D EPR spectroscopy were not successful due to strong gamma ray absorption by tungsten and intramolecular magnetic interactions, respectively.

To gain insight into the catalysis by TBA{SiFe_3_W_9_} and the promoting effects of alkali cations, a theoretical analysis was carried out by combining classical molecular dynamics (MD) simulations and static density functional theory (DFT) calculations on the combination of the polyoxometalate anion, {SiFe^III^_3_W_9_O_37_^7–^}, LiClO_4_, and TBA^+^ in THF.

**Figure 5:**
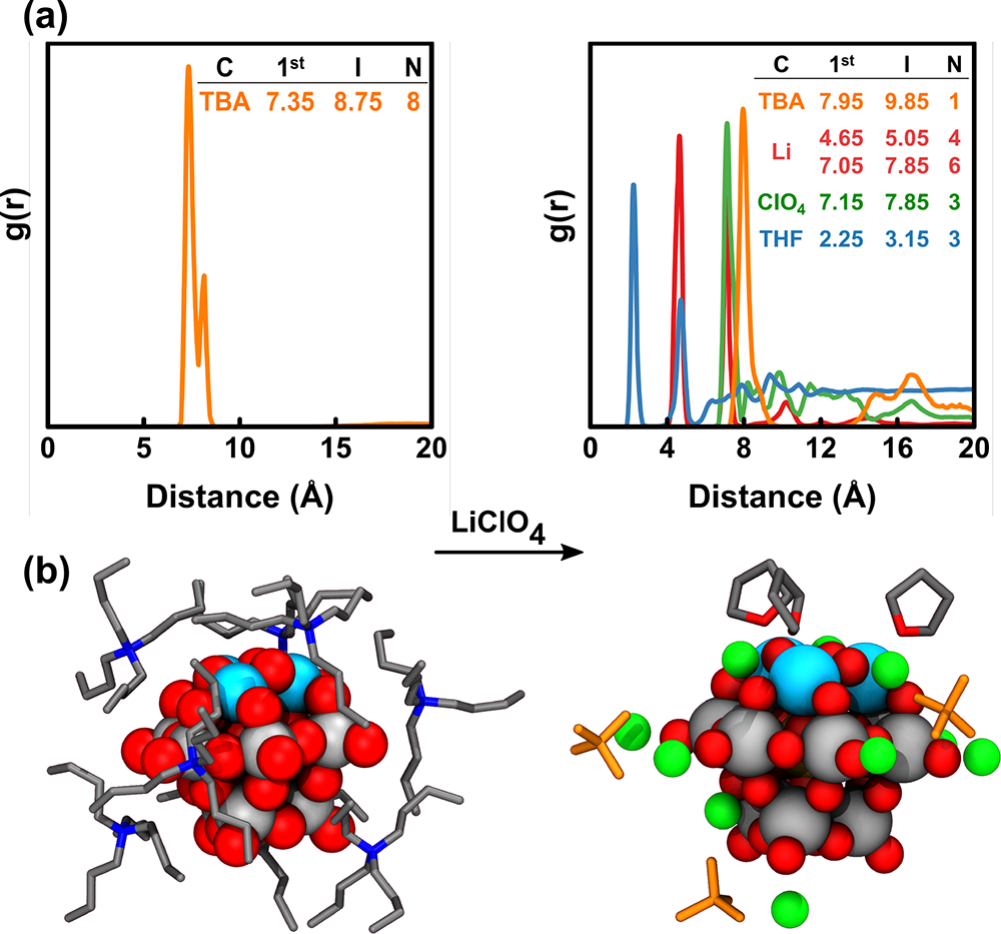
Classical Molecular Dynamics (MD) Simulations. (a) Combined radial distribution functions (RDFs) between {SiFe^III^_3_W_9_O_37_^7–^}···TBA (orange), {SiFe^III^_3_W_9_O_37_^7–^}···Li (red), {SiFe^III^_3_W_9_O_37_^7–^}···ClO_4_^–^ (green), and {SiFe^III^_3_W_9_O_37_^7–^}···THF (blue), taking as references the central Si atom of {SiFe^III^_3_W_9_O_37_^7–^}, the N atom of TBA, Li, the Cl atom of ClO_4_^–^, and the O atom of THF. The first peak(s) (first), the integration distance (I), both in Å, and the coordination number (N) are given. In the absence of LiClO_4_, eight TBA cations surround {SiFe^III^_3_W_9_O_37_^7–^}, but after the addition of LiClO_4_, they are shifted from 7.35 to 7.95 Å. The first two {SiFe^III^_3_W_9_O_37_^7–^}···Li peaks show two Li^+^ coordination sites: the first one (4.65 Å) closer to bridging oxygens and the second one (7.05 Å) closer to terminal oxygens. See Figure S5 for more details. (b) Representative snapshots of the coordination sphere of {SiFe^III^_3_W_9_O_37_^7–^} without (left) and with (right) LiClO_4_. Fe – Light blue; O – red; N – blue; W – gray; C – silver; ClO_4_^–^ – orange; and Li – green.

MD results show that upon inclusion of LiClO_4_ into the model the TBA^+^ counterions initially randomly distributed around {SiFe^III^_3_W_9_O_37_^7–^} move away while the Li^+^ cations approach {SiFe^III^_3_W_9_O_37_^7–^}, Figure 5a. The radial distribution function (RDF) of a 20 ns MD run shows a narrow peak at 4.65 Å assigned to four Li^+^ cations in contact with bridging oxygen atoms of {SiFe^III^_3_W_9_O_37_^7–^}. As expected from the molecular electrostatic potential distribution in Figure S5, three of these Li^+^ cations are attracted by the distinctly basic Fe^III^_3_O_3_ unit, forming a strongly coordinated Li_3_Fe^III^_3_O_3_ moiety. At 7.05 Å, another narrow peak appears integrating to 6.33, associated with Li^+^ cations weakly interacting with terminal oxygens of {SiFe^III^_3_W_9_O_37_^7–^}. Moreover, three ClO_4_^–^ ions were found near the Li^+^ cations, leading to a neutral moiety formulated as {SiFe^III^_3_W_9_O_37_^7–^}/10 Li^+^/3 ClO_4_^–^/3 THF, a snapshot of which, formed after 20 ns, is given in Figure 5b. After the interaction of Li^+^ with {SiFe^III^_3_W_9_O_37_^7–^} three THF molecules can be found at an average distance of 2.25 Å (2.13 Å upon DFT optimization) to Fe^III^. Before the addition of LiClO_4_, no THF is bound to Fe^III^ atoms.

The computed interaction between TBA{SiFe_3_W_9_} and Li^+^ was verified by ^7^Li NMR spectroscopy, where both the 1D and the diffusion coefficients of ^7^Li^+^ in the presence and absence of {SiFe_3_W_9_} were compared, Figure S6. The results show first that there is a fast exchange between bound and unbound Li^+^ in the presence of {SiFe_3_W_9_} accompanied by an upfield shift of the ^7^Li peak versus a solution containing Li^+^ only. Second, there is a decrease of ∼50% in the diffusion constant in the presence of {SiFe_3_W_9_}, which in the control experiment was shown to be unrelated to a change in the solution viscosity, thereby providing experimental support for the binding contact of Li^+^ to TBA{SiFe_3_W_9_}.

**Figure 6:**
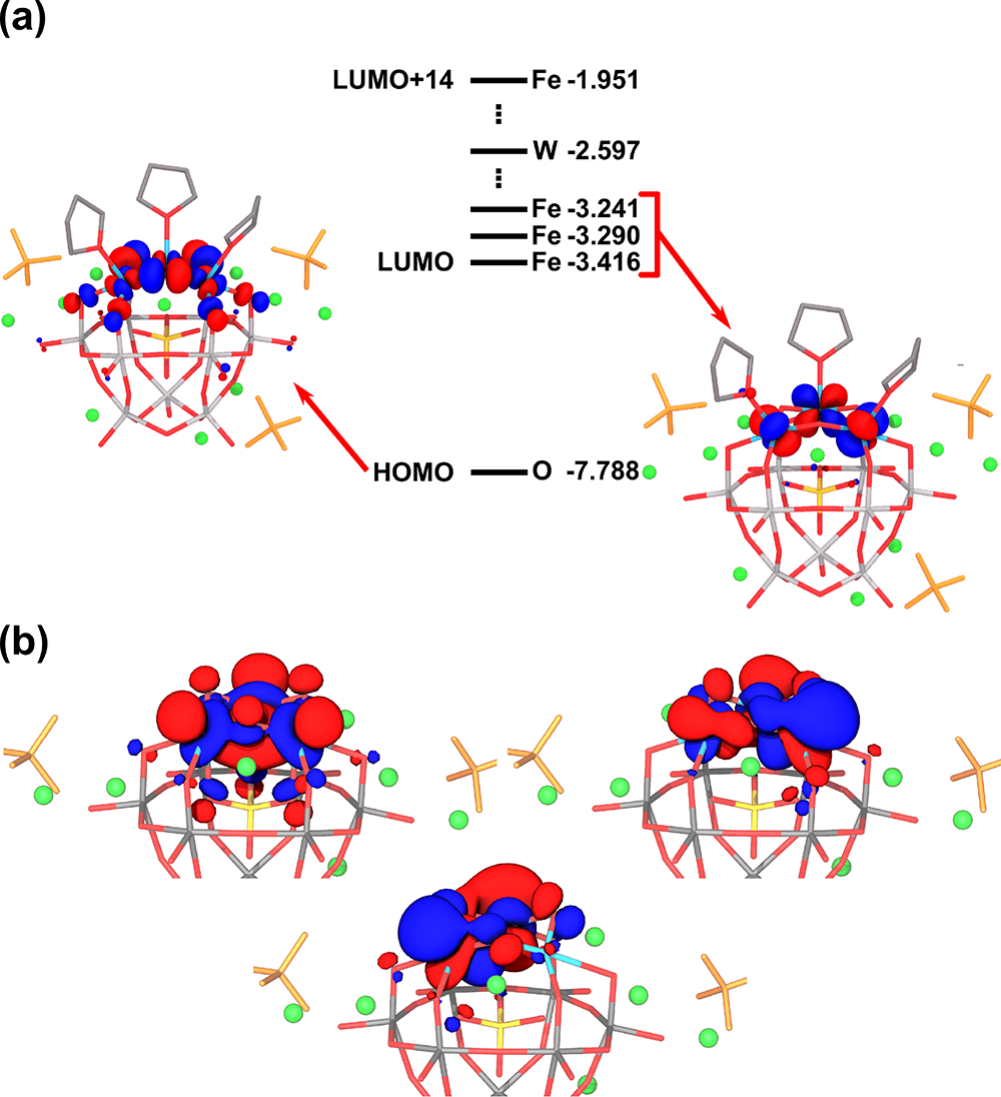
Frontier molecular orbitals (MO) of {SiFe^III^_3_W_9_O_37_^7–^}/10 Li^+^/3 ClO_4_^–^/3 THF. (a) Diagram showing the HOMO and LUMOs of {SiFe^III^_3_W_9_O_37_^7–^}/10 Li^+^/3 ClO_4_^–^/3 THF embedded in a continuum dielectric. Energies are in eV. Fe, W, and O labels indicate the atom with the highest contribution to each MO. Delocalized W-like orbitals are inserted in the set of Fe MOs, allowing W centers to act as electron reservoirs. LUMO and HOMO representations are given, with no significant differences in shape of the other two Fe-centered LUMOs. (b) Representation of the three MOs occupied upon the 3e^–^ reduction, showing the formation of three Fe^II^ centers. Distribution of the spin density is given in Figure S7.

A total description of the environment around polyoxometalates is crucial for the truthful determination of molecular orbital (MO) energies. Introducing the combined effect of counterions and solvent molecules with the dielectric continuum model only, as typically done for the analysis of electrochemical properties of polyoxometalates,^49^ would not reveal the real influence of Li^+^, as it has been recently shown in the calculations of redox potentials associated with proton coupled electron transfer (PCET) events.^50,51^ Thus, the electronic properties of {SiFe^III^_3_W_9_O_37_^7–^}/10 Li^+^/3 ClO_4_^–^/3 THF were explored by using DFT calculations. Magnetic susceptibility measurements, Figure S8, in solution using the Evans method^52^ showed that both {SiFe^III^_3_W_9_} and {SiFe^II^_3_W_9_} were high spin compounds with approximately 15 and 12 unpaired electrons, respectively. Accordingly, the calculations were carried out considering high spin species. The remaining components of the solution were modeled using a continuum dielectric approach. The importance of adding explicit cations in the present calculations is clear; there is an energy difference of more than 3 eV between the lowest unoccupied orbital of {SiFe^III^_3_W_9_O_37_^7–^} computed as a single anion using PCM as the continuum model and as {SiFe^III^_3_W_9_O_37_^7–^}/10 Li^+^/3 ClO_4_^–^/3 THF and the PCM model, Figure S9. The presence of Li^+^ stabilizes the molecular orbitals of {SiFe^III^_3_W_9_O_37_^7–^} so that reduction occurs at moderate potentials. A schematic MO diagram for {SiFe^III^_3_W_9_O_37_^7–^}/10 Li^+^/3 ClO_4_^–^/3 THF is shown in Figure 6a. Expectedly, the three lowest unoccupied orbitals are located at the three Fe atoms.

However, when three electrons are added to the system, two of them localize at Fe atoms, whereas the third is delocalized among several W atoms (Figure S10). Still, an important consequence of the reduction is that the computed Fe–O_THF_ bond lengths increase from about 2.13 Å in the fully oxidized POM to values between 2.21 and 2.36 Å in the 3-electron reduced one. As a result, there is a dramatic decrease in the binding energy for the three THF ligands, from −50 to only −8 kcal mol^–1^ upon the addition of three electrons. Thus, it is reasonable to think that upon incorporation of entropic effects, the decoordination of THF ligands becomes thermodynamically favorable. Importantly, in the absence of THF ligands, all three extra electrons are accommodated on the Fe centers (Figure 6b), forming a {SiFe^II^_3_W_9_} complex, as previously proposed by Pope and co-workers.^48^ It is important to note that the deligation of THF upon reduction is an essential requirement for coordination of N_2_ to the reduced center in the electrocatalytic reaction.

**Figure 7:**
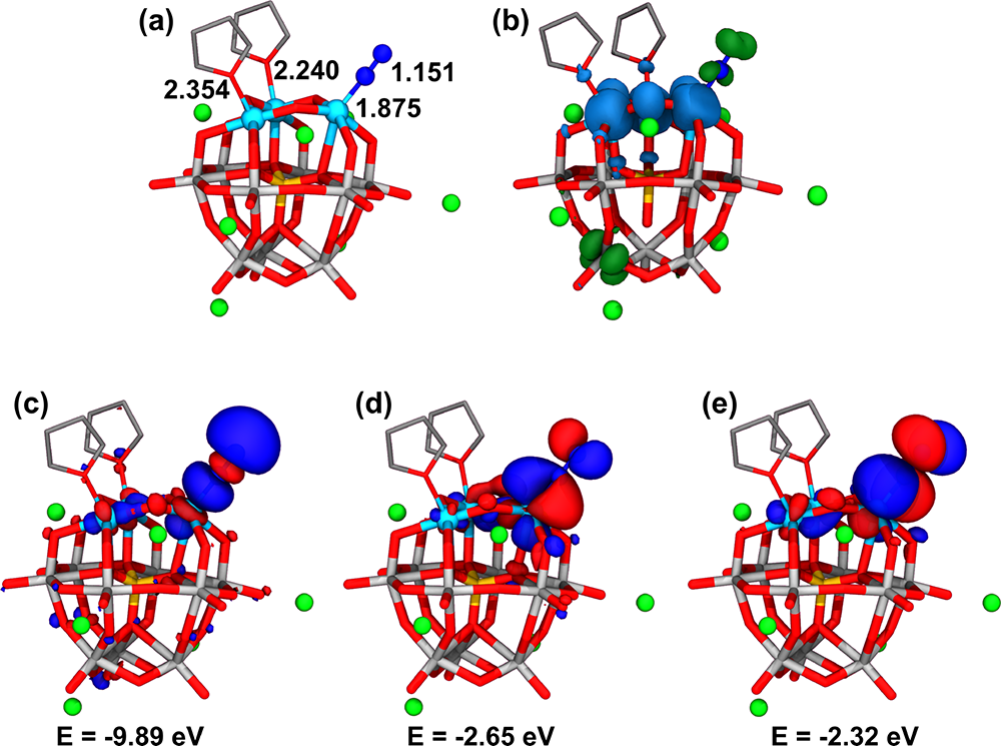
Representation of (a) the coordination of N_2_ to the polyoxometalate, (b) the spin density distribution (blue and dark green surfaces for excess of α- and β-spin electron density, respectively) on the structure shown in panel (a); and (c–e) the main molecular orbitals involved in the interaction between Fe and N_2_. The binding of N_2_ was calculated using the three-electron reduced species, {SiFe^III^_3_W_9_O_37_^10–^}/10 Li^+^/2 THF. Selected bond lengths are in Å, and MO energies are in eV.

Additional DFT calculations were conducted to investigate the nature of the interaction between the reduced catalyst and N_2_. Surprisingly, all attempts to coordinate N_2_ to the ligand-free model of the reduced catalyst were unsuccessful, as the N_2_ molecule spontaneously decoordinates the Fe center during the geometry optimization. However, when considering a concerted coordination of N_2_ to one Fe^II^ center together with the association of 2 THF solvent molecules to the two remaining ones, the structure represented in Figure 7 was obtained (for more details see Figures S11 and S12). The latter shows that N_2_ can bind to an Fe center to form an Fe–N bond with a bond length of 1.87 Å and a slightly elongated N–N bond distance of 1.15 Å relative to N_2_ (1.10 Å), Figure 7a. The σ and π molecular orbitals arising from Fe–N_2_ bond formation are shown in Figure 7c and d–e, respectively. The π-type orbitals result from the combination of the d_*xz*_ and d_*yz*_ orbitals of the metal with the π* orbitals of N_2_ and were found to host one spin-down electron each. The third extra electron in this structure was found to be delocalized over the polyoxotungstate framework. Thus, this species might be interpreted as an Fe^II^–N_2_^•–^ complex in which the extra electron supported by the N_2_ moiety is antiferromagnetically coupled to the *d*-electrons of the metal ion (Figure 7b). Classically, this binding mode may be seen as an Fe^I^–N_2_ complex whereby the N_2_ molecule is activated via π back-donation from the d orbitals of the electron-rich Fe center to the empty π* orbitals of N_2_.

Overall, these results suggest that, despite being weak, the “*pushing*” electron-donating effect of THF solvent molecules is required to destabilize d(Fe) orbitals, shifting them up in energy above the π-type molecular orbitals shown in Figure 7d–e, thus promoting the activation of N_2_. The latter requires two additional electrons (Figure 7d–e), indicating that the cooperative effect of an Fe binding site and a second redox-active center acting as an electron reservoir is needed to activate N_2_.

**Figure 8:**
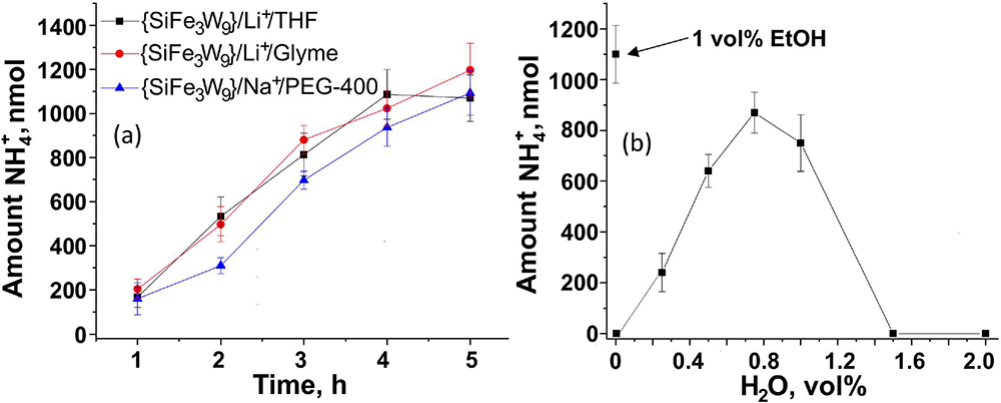
Controlled Potential Electrolysis. Left - CPE at −1.8 V versus Ag/AgCl (−2.4 V versus SHE, Figure S4) in THF and glyme were carried out in 10 mL of dry solvent containing 0.1 M TBAPF_6_, 0.5 mM TBA{SiFe_3_W_9_}, 25 mM LiClO_4_ with 1 vol % (0.17 M) ethanol as proton donor under 1 bar N_2_ using a copper wire working electrode, a platinum wire counter electrode, and a Ag/AgCl reference electrode. Right - CPE measurements −1.8 V versus Ag/AgCl (−1.3 V versus SHE, see Figure S14) were also carried out in PEG-400 using water as proton/electron donor in 10 mL of PEG-400 containing 0.1 M TBAPF_6_, 0.5 mM TBA{SiFe_3_W_9_}, and 25 mM NaClO_4_ with varying amounts of water under 1 bar of N_2_ for 5 h using a copper wire working electrode, a platinum wire counter electrode, and a Ag/AgCl reference electrode. The results presented are an average of three experiments, and the error bars define the range. 1 vol % H_2_O is 0.56 M. The faradaic efficiencies as a function of time are given in Table S5 and are typically ∼8%. In PEG and in the absence of TBA{SiFe_3_W_9_}, there was only a very low current of ∼1 μA, Figure S15, and no NH_3_ formation, while for the reaction with TBA{SiFe_3_W_9_} the current was an order of magnitude higher.

Controlled potential electrolysis (CPE) of a 10 mL solution using a dried solvent (THF, glyme) containing 0.1 M TBAPF_6_, 0.5 mM TBA{SiFe_3_W_9_}, and 25 mM LiClO_4_ with 1 vol % ethanol as a proton donor under 1 bar of N_2_ at −1.8 V versus Ag/AgCl (∼2.4 V SHE) using a 1 cm long × 1 mm diameter copper wire cathode (surface area ∼0.3 cm^2^), and a platinum wire anode separated by a frit showed the formation of NH_3_ in increasing amounts over time in both THF and less volatile glyme as solvent with 1 vol % ethanol as a proton donor, Figure 8a. No formation of H_2_ or NH_2_NH_2_ was detected. No ammonia was detected using He as a control.

In order to provide additional confirmation of formation of NH_3_ from N_2_, parallel experiments were carried out in an electrolyzer (see Experimental Section for details) using a 0.25 cm^2^ copper foil cathode. Reaction with ^14^N_2_ or with purified ^15^N_2_ (98% labeled) showed the formation of 760 nmol of ^14^NH_3_ and 740 nmol of ^15^NH_3_ after 5 h in high isotopic purity, Figure S13. No NH_3_ formation was observed with TBA{SiFe_3_W_9_} or Li^+^ alone, noting also that the Li^0/1+^ redox couple is at −3.0 V versus SHE. No deposition of Li metal or LiO_2_ on the cathode was detected using ICP-MS.

From an applicative/commercial point of view of electrocatalytic N_2_ reduction, water would be the preferred source of protons and electrons via an anodic water oxidation reaction. In addition, lithium is likely to be supply limited and electrocatalytic formation of NH_3_ from N_2_ using Na^+^ as a promoter would also be advantageous. NaClO_4_ is only slightly soluble in THF. Thus, polyethylene glycol (PEG-400), known to coordinate alkali metal cations via an induced dipole interaction,^53^ was used as solvent to solubilize NaClO_4_. After confirming that there was no NH_3_ formation under He, this combination, TBA{SiFe_3_W_9_}, NaClO_4_, and PEG-400, with ethanol as the proton donor yielded NH_3_ with a rate of formation that was similar to what was observed using TBA{SiFe_3_W_9_}, Li^+^, THF, and ethanol, Figure 8a. Although the combination of TBA{SiFe_3_W_9_}, Li^+^, THF, or glyme was inactive with water as the proton and electron donor, addition of water instead of ethanol, up to 1 vol %, to TBA{SiFe_3_W_9_}, Na^+^ in PEG-400 showed that water can be used as the electron and proton donor for NH_3_ formation, Figure 8b. The optimum amount of H_2_O for NH_3_ formation was 0.75 vol % (∼0.42 M) allowing the use of water as the proton and electron donor for e-N2RR through its oxidation at the anode with coformation of O_2_, Figure 1. The optimum potential appeared to be −1.3 V versus SHE, with no reaction at less negative potentials and surprisingly no significant improvement at −1.5 V. The ^15^N_2_ experiment using a 0.25 cm^2^ copper foil cathode in reactions with ^14^N_2_ or with purified ^15^N_2_ (98% labeled) showed the formation of 840 nmol ^14^NH_3_ and 920 nmol ^15^NH_3_ after 5 h with a *very* high isotopic purity, Figure S16.

It should be noted that PEG-400 is in itself an alcohol and thus can in principle act as a proton and electron donor, although in the absence of water no NH_3_ was formed; see Figure 8b entry of 0% water. To further verify that water in fact is the source of protons and electrons, a further reaction was carried out using the dimethyl ether of PEG-400 as solvent in the presence of 1 vol % water. Thus, 10 mL of PEG-400-Me_2_ containing 0.1 M TBAPF_6_, 0.5 mM TBA{SiFe_3_W_9_}, and 25 mM NaClO_4_ with 1.0 vol % water under 1 bar of N_2_ for 5 h at −1.3 V versus SHE using a copper wire working electrode, a platinum wire counter electrode, and a Ag/AgCl reference electrode yielded a solution containing 900 nmol of NH_3_, essentially the same result that was obtained using PEG-400 as solvent.

In addition, taking advantage of the ability of PEG to coordinate alkali metal cations, the reaction was further optimized using the potassium salt of the catalyst, K{SiFe_3_W_9_}, and sodium triflate as electrolyte. Thus, in a simple undivided cell electrolyzer, consisting of a 0.25 cm^2^ Cu foil cathode, a stainless-steel anode loaded with 2 mL of PEG-400 containing 0.5 mM Na{SiFe_3_W_9_}, 1 vol % H_2_O, and 0.1 M NaCF_3_SO_3_ under 1 bar of N_2_ operated at −1.3 V versus SHE yielded 900 nmol of NH_3_ after 3 h (0.35 nmol sec^–1^ cm^–2^) with a faradaic efficiency of 24%. See the Conclusions section for a discussion on the faradaic efficiency and overpotential.

To support the overall premise that the N_2_ to NH_3_ reduction indeed occurs on or is initiated at the cathode, a higher intrinsic surface area Cu foam cathode with a geometric surface area of 0.13 cm^2^ yielded 1600 nmol of NH_3_ or 1.15 nmol sec^–1^ cm^–2^. See Figure S17 for a current versus time plot showing a steady current over time. A similar experiment (2 mL of PEG-400 containing 0.5 mM K{SiFe_3_W_9_} and 0.1 M NaCF_3_SO_3_ under 1 bar of N_2_ operated at −1.3 V versus SHE) with a 0.13 cm^2^ Ni mesh cathode, noncorrosive to NH_3_, produced 1400 nmol of NH_3_ at a rate of 1.0 nmol sec^–1^ cm^–2^ with a faradaic efficiency of 25%. See Figure S18 for a current versus time plot showing a slight increase in current over time. The experiments on both the Cu foam and Ni mesh yielded 1.6 and 1.4 turnovers, respectively, relative to the amount of catalyst in solution. See the discussion below related to the intrinsic catalyst active on the electrode. An additional 10 h experiment using 2 mL of PEG-400 containing 0.5 mM Na{SiFe_3_W_9_}, 1 vol % H_2_O, and 0.1 M NaCF_3_SO_3_ under 1 bar of N_2_ operated at −1.3 V versus SHE yielded 5100 nmol of NH_3_ (5.1 turnovers relative to the amount of Na{SiFe_3_W_9_} in solution after 10 h (1.09 nmol sec^–1^ cm^–2^) with a faradaic efficiency of 27%). See Figure S19 for the current versus time plot that shows a stable current over time.

Various additional experiments were carried out (1) to verify the required presence of iron centers in the catalyst, (2) to obtain a sense of the importance of the N_2_ pressure given its low solubility, and (3) some initial experiments showing catalyst stability:

(1) Carrying out a reaction using Na_4_SiW_12_O_40_ instead of Na{SiFe_3_W_9_} (2 mL of PEG-400 containing Na_4_SiW_12_O_40_, 1 vol % H_2_O, and 0.1 M NaCF_3_SO_3_) under 1 bar N_2_ operated at −1.3 V versus SHE for 3 h yielded no formation of NH_3_ whereas Na{SiFe_3_W_9_} yielded 1300 nmol of NH_3_ leading to the conclusion that Fe sites are required active sites for catalysis.^54^
(2) Comparison reactions under different N_2_ pressures were carried out in an undivided cell placed in a Büchi mini autoclave. A solution of 5 mL of PEG-400 containing 0.1 M NaSO_3_CF_3_ as the electrolyte and 0.5 mM K{SiFe_3_W_9_} with a 0.25 cm^2^ copper foam cathode, a stainless-steel mesh anode, and a Ag/AgCl reference electrode was reacted for 1 h under 1, 30, or 50 bar of N_2_, yielding 20, 115, or 165 nmol of NH_3_, respectively; see Figure S20. Clearly, the reaction rate is a function of the N_2_ pressure and concentration. Therefore, rates of ∼10 nmol s^–1^ cm^–2^ are attainable through an increase in pressure even on a low surface cathode.
(3) Several experiments were carried out to see if any species were deposited on the cathode indicating significant catalyst instability or if active catalytic species were deposited on the cathode leading to a heterogeneous catalyst instead of {SiFe_3_W_9_} as a molecular catalyst. Using an undivided cell electrolyzer, consisting of a 0.25 cm^2^ Cu foil cathode, a stainless-steel anode loaded with 2 mL PEG-400, 0.5 mM Na{SiFe_3_W_9_}, 1 vol % H_2_O, and 0.1 M NaCF_3_SO_3_ under 1 bar of N_2_ operated at −1.3 V versus SHE yielded ∼900 nmol of NH_3_ after 3 h as described above. (a) The cathode was gently washed with DDW and then treated with DDW with sonication. ICP-MS analysis did not show the detectable presence of Fe or W. (b) Similarly, after removal of the cathode after 2 h and a gentle wash, the reaction was continued with the same cathode for another 2 h. Figure S21 shows the result of the experiment where only a small change in current was observed, probably associated with a loss of a small amount of catalyst in the wash procedure. Almost equal amounts of ∼650 nmol of NH_3_ were formed in each 2 h reaction. (c) In a further experiment, the cathode was removed from the reaction mixture after 5 h and used in a fresh reaction solution that did not contain any Na{SiFe_3_W_9_}. No measurable amount of NH_3_ was formed. (d) Longer reaction times are not feasible in an undivided (membrane-less) cell set up using H_2_O as the proton/electron donor, leading to an undesired pH gradient at the cathode^55^ and formation of NH_4_^+^ in solution. The NH_4_^+^ metathetically exchanges the Na^+^ cation in Na{SiFe_3_W_9_}. {SiFe_3_W_9_} with even only two NH_4_^+^ cations has a low solubility constant and leads to its gradual precipitation. In order to remediate this problem, we have carried out a reaction in a 2 cm × 2 cm electrolyzer consisting of a stainless-steel cathode, a titanium felt anode, and with a Zirfon Perl UTP 500 membrane to separate between the cathode and anode. The electrolyzer cell contained 2 mL of PEG-400 with 1 vol % H_2_O, 0.5 mM K{SiFe_3_W_9_}, and 0.1 M NaCF_3_SO_3_. A reaction was carried out for 24 h under a constant flow of purified and recycled N_2_ yielding 98 μmol of NH_3_ captured ex-situ by an acid trap. As can be seen from Figure S22, the current is stable during the reaction period at 70–72 mA.

It would appear that the N_2_ reduction reaction is initiated at the cathode and not in solution. The extraction of kinetic parameters using a rotating disc electrode is a topic for future research. However, already now it would still be useful to get a rough estimate of the intrinsic catalytic activity of {SiFe_3_W_9_} taking into account a reversible electron transfer of adsorbed {SiFe_3_W_9_} on the cathode, Figure S1, and the overall observation that rates of formation of NH_3_ are higher on a Cu foam cathode (1.15 nmol sec^–1^ cm^–2^) versus a Cu foil cathode (0.35 nmol sec^–1^ cm^–2^). The cross-section of {SiFe_3_W_9_} is ∼2 nm from the MD calculations and, thus, occupies an area of 3.14 nm^2^. Arbitrarily, assuming a 10% coverage of {SiFe_3_W_9_} on the cathode, the number of molecules on the surface would be ∼8 × 10^12^ molecules or ∼1.3 × 10^–3^ nmol. Assuming the linear formation of NH_3_ over time, Figure 8, the intrinsic turnover frequency (TOF) of the reaction catalyzed by {SiFe_3_W_9_} would be 64 s^–1^. This TOF is 30 times higher than the TOF of the nitrogenase enzyme.^5^ Even with monolayer formation, that is, 100% coverage on the cathode, the intrinsic TOF would still be a rather respectable 6.4 s^–1^

## Conclusions

In the first phase of the research, a tri-iron substituted polyoxometalate, TBA_7_[Fe_3_(H_2_O)_3_SiW_9_O_37_], was shown to bind Li^+^ cations to oxygen atoms mostly in the vicinity of the more basic tri-iron moiety. This interaction, shown experimentally by CV and ^7^Li NMR measurements and computationally by MD simulations, leads to the decrease in the reduction potential of the polyoxometalate, allowing the coordination and activation of N_2_ in THF at about −1.9 V versus SHE as demonstrated by UV–vis measurements. Additional CV measurements in the presence of ethanol as a proton/electron donor showed a catalytic wave with an onset potential of 1.85 V versus Fc/Fc^+^ and a reversible electron transfer.

Building on the UV–vis measurements showing N_2_ activation, CPE was carried out based on the protocols developed for such experiments,^29,30^ requiring very high purity N_2_ to eliminate NO_*x*_ gas contamination, null product formation in reaction with inert He combined with stringent ^15^N_2_ labeled experiments to rule out contamination by nitrite or nitrate anions. Reactions were first carried out under anhydrous conditions in THF in the presence of TBA{SiFe_3_W_9_} and Li^+^ using ethanol led to formation of 1+μmol amounts of NH_3_ after 5 h at −2.4 V versus SHE. No hydrazine or hydrogen formation was observed in this and ensuing CPE reactions. Importantly, ^15^N_2_ experiments confirmed the lack of significant ^14^N contamination from any source. Buoyed by this result, further experiments catalyzed by TBA{SiFe_3_W_9_} were carried out in PEG-400 using Na^+^ as the activating cation instead Li^+^ and either ethanol or water as the electron and proton source. At up to 1 vol % water and at −1.3 V versus SHE, the electrocatalytic formation of NH_3_ was successful in yields similar to those found in the anhydrous medium using ethanol as the proton and electron donor. The ^15^N_2_ experiments carried out in PEG-400 with H_2_O as the proton and electron donor also confirmed the lack of any ^14^N contamination from any source.

An additional advantage of using PEG-400 as solvent is that a potassium salt of the polyoxometalate, K{SiFe_3_W_9_}, can be used as catalyst. In an undivided cell electrolyzer configuration using a Cu foil cathode and water as a proton and electron donor at −1.3 V versus SHE, NH_3_ was formed at a constant current and at a rate of 0.35 nmol sec^–1^ cm^–2^ with a faradaic efficiency of 24%. Based on the premise that the N_2_ is reduced to NH_3_ at the surface of the cathode (demonstrated by using a Cu foam electrode) and not in solution, higher reaction rates of >1 nmol sec^–1^ cm^–2^ were obtained. The intrinsic activity of {SiFe_3_W_9_}, a TOF of 64 s^–1^, could be roughly calculated assuming an arbitrary coverage of 10% on the smooth copper surface. Since copper is corrosive to NH_3_ an additional experiment using a Ni mesh electrode similarly yielded 1 nmol sec^–1^ cm^–2^. Longer reaction times of 10 h using a Cu foam cathode led to the formation of 5.1 equiv of NH_3_ per equivalent of {SiFe_3_W_9_}. Together with this result, various control reactions and chronoamperometric measurements provide satisfactory and preliminary evidence of good catalyst stability.

These initial results on this new catalytic system need to be further developed to understand its true potential. This will require experimentation in a divided cell membrane electrode assembly with high surface area gas diffusion electrodes for a much more efficient mass transfer of N_2_ at the cathode in order to obtain higher current densities. Presently in our two-electrode undivided cell configuration, the faradaic efficiencies, also an important parameter, are still moderate, typically 25–30%. No other products, notably, NH_2_NH_2_ or H_2_, were observed in the CPE reactions. Given that the {SiFe_3_W_9_} catalyst is redox reversible and present at the cathode and anode, the working hypothesis is that the moderate faradaic efficiencies observed are mainly due to the fact that in an undivided cell configuration reduced {SiFe_3_W_9_} or intermediate species therefrom can be reoxidized at the anode without a net formation of NH_3_. It should be stressed here that the basis for this hypothesis is the well and long-known very fast outer sphere electron transfer between polyoxometalates. For example, electron self-exchange for Keggin type anions is typically in the range of 10^5^ to 10^7^ M^–1^ s^–1^,^56^ thereby allowing the through solution (back) transfer of electrons from the cathode to the anode driven by the positive potential, >+1.2 V at the anode. A divided cell membrane electrode assembly with no catalyst at the anode will allow true evaluation of the possible faradaic efficiency in a realistic setting. It will also allow pH control to prevent formation of NH_4_.^55,57^ Such a membrane electrode assembly setup will probably allow the increase in the rates of NH_3_ formation from N_2_. In fact, initial experiments, Cu foam versus Cu foil cathodes and suitable N_2_ pressures, already indicate that properly designed high surface area cathodes could lead to practical interesting rates of NH_3_ formation using anodic water oxidation as the source of electrons and protons at low potentials of −1.3 V versus SHE. Finally, although the potential at the cathode is only −1.3 V vs SHE, the overpotential calculated assuming pH = 7 in PEG-400 is 0.95 and 0.815 V at pH = 10. The cathodic and also anodic overpotentials are exothermic, and therefore they will lead to an increase in temperature. However, the cathodic exothermicity is too low, estimated at 2–2.5 cal h^–1^, to easily measure considering both the amount of NH_3_ formed and the faradaic efficiency.^58^

## Experimental Section

### Instruments

Electrochemical experiments were carried out using a Biologic multichannel VSP 201 potentiostat. Gas phase analysis of H_2_ was carried out using a GOW MAC gas chromatograph, with a thermal conductivity detector and two columns in series (4’X1/8’’ St. St. Hayesep T, 10’X1/8’’ St. St., Molecular sieve SA) with Ar as a gas carrier. UV–vis measurements were done using an Agilent 8453 UV–visible spectrometer with deuterium and tungsten lamps as light sources. IR measurements were carried out on a Nicolet 5700 FTIR instrument. Mass spectrometry measurements were done with a Xevo G2-XS QTOF high resolution ESI TOF MS instrument. NMR measurements were done with a Bruker AVANCE III HD-500 MHz magnet. Thermo gravimetric analysis was measured using an SDT Q 600 using alumina crucibles. ICP-MS analysis was carried out using an Agilent 7700s spectrometer.

### Solvents, Gases, and Chemicals

Tetrahydrofuran (THF), 99.9%, water <50 ppm, purchased from Acros Organic, was additionally dried over molecular sieve 4 Å and stored in a glovebox. Polyethylene glycol (PEG) 400 grade extra pure was further distilled to remove water by an azeotropic distillation with toluene. 1,2-Dimethoxyethane (DME) extra pure 99% was purchased from Fischer Chemical and was further purified by reflux distillation under vacuum and further dried over molecular sieve 4 Å.^59^ The purified solvents were stored in a glovebox. Ethanol (EtOH) 99.9% dried over molecular sieve 4 Å and dimethyl sulfoxide (DMSO-d6) were purchased from Merck Millipore. Dichloromethane (DCM), sodium hypochlorite 6%, hydrochloric acid (HCl) 37%, and sulfuric acid (H_2_SO_4_) 98% were purchased from Biolab. Anhydrous sodium carbonate 99.5%, tetra *n*-butyl ammonium (TBA) hydrogen sulfate, and tetra *n*-butyl ammonium PF_6_ (TBAPF_6_) were purchased from Acros. Sodium metasilicate, sodium tungstate, sodium acetate trihydrate, potassium chloride, sodium chloride, iron(III) nitrate nonahydrate, phenol 99%, ferrocene 98%, and ferrocenium hexafluorophosphate 97% were purchased from Sigma-Aldrich. LiClO_4_ trihydrate, hydrazine monohydrate 99%, and 4-dimethylaminobenzaldehyde were purchased from Alfa Aesar, and NaClO_4_ was purchased from Fluka AG. Sodium nitrosopentacyanoferrate (sodium nitroprusside) was purchased from BDH Limited Poole England. Sodium hydroxide pearls were purchased from Biolab, and ammonium hexafluorophosphate was purchased from Apollo Scientific. Helium 99.999% and Nitrogen 99.9999% were purchased from Maxima Ltd., and N_2_ was further purified using a VICI NPM-220 nitrogen mini-purifier which at a minimum reduces N impurities by 2 orders of magnitude. The below detection limits of NO_*x*_ compounds in He and after purification of ^14^N_2_ and ^15^N_2_ were verified by GC-MSD and using Griess spectrometry; the nitrate/nitrite colorimetric assay kit was purchased from Cayman Chemical. Isotopically labeled nitrogen ^15^N_2_ 98%, also purified using a VICI NPM-220 nitrogen mini-purifier, and ammonium sulfate, ^15^N_2_ 98%, were purchased from Cambridge Isotope Laboratories, Inc. Water (∼18 mΩ/cm^–1^) was used throughout all the experiments.

### Sodium α-Nonatungstosilicate, Na_10_[α-SiW_9_O_34_]·H_2_O

Na_10_[α-SiW_9_O_34_]·H_2_O was synthesized according to literature procedure.^60^ Thus, Na_2_WO_4_·2H_2_O (550 mmol) and Na_2_SiO_3_ (50 mmol) were dissolved in 50 mL of hot water (90 °C) in a 250 mL beaker. To this solution were added dropwise 32.5 mL of 6 M HCl during ∼30 min with vigorous stirring. The solution was boiled until the volume reaches ∼75 mL, the solution was cooled down, and unreacted silica was removed by filtration over a sintered glass frit. Anhydrous sodium carbonate (12.5 g) dissolved in 37.5 mL water was added slowly to the previously filtered solution. The initial precipitate was removed by filtration using Whatman 90 mm filter paper. Then the solution is left under stirring for 1 h, and afterward 250 mL of 4 M NaCl were added and the precipitate obtained was collected over a sintered glass frit and washed successively with 25 mL of ethanol and 25 mL of diethyl ether and then dried under vacuum. Yield 85%. Na_10_[α -SiW_9_O_34_]·H_2_O was characterized by IR using a 2–5% compound in a KBr pellet.

### Synthesis of the α-[Si{Fe^III^(H_2_O)}_3_W_9_O_37_]^−7^ as a Tetra-*n*-butyl Ammonium Salt, TBA{SiFe_3_W_9_}

The synthesis of K_7_[SiFe^III^_3_(H_2_O)_3_W_9_O_37_], K{SiFe_3_W_9_}, was done according to literature procedure as follows:^48^ A solution of sodium acetate trihydrate (pH 6.5, 200 mL) was added slowly to a solution of iron(III) nitrate (53 mmol) in 100 mL of water. The color changed from brown to deep red (pH ∼ 4). Afterward, the solution was heated to 80 °C and Na_10_[α -SiW_9_O_34_]·H_2_O (1.6 mmol) was added over a 1 h period with vigorous stirring. After the addition was complete the solution was left with gentle stirring for 1 h at 80 °C. The solution was the cooled down and filtrated using Whatman 90 mm filter paper. The filtrate was treated with 6.5 g of KCl in 25 mL. The precipitate was collected over filter paper and air-dried. Yield 50%. K_7_[SiFe^III^_3_(H_2_O)_3_W_9_O_37_] was characterized by IR using 2–5% compound in a KBr pellet, Figure S23. The molecular purity was verified by high resolution mass spectrometry using a Xevo G2-XS QTOF high resolution ESI TOF mass spectrometer; see Figure S24. The potassium cation was exchanged with a tetra-butylammonium cation (TBA) as follows: K_7_[Si{Fe(H_2_O)}_3_W_9_O_37_] (1 mmol) was dissolved in 50 mL of water, and separately tetra-*n*-butyl ammonium hydrogen sulfate (100 mmol) was dissolved in dichloromethane (50 mL). Both solutions were transferred to a separatory funnel, which then was shaken vigorously several times, and the lower organic phase was extracted and evaporated to dryness. The TBA ratio to the α-[Si{Fe(H_2_O)}_3_ W_9_O_37_] anion ratio was determined using TGA, Figure S25.

### Synthesis of K_10_[SiFe^II^_3_(H_2_O)_3_W_9_O_37_]

The synthesis was carried out in a glovebox by preparing a solution of 261 mg of Fe(CH_3_CO_2_)_2_ in 25 mL of TDW 0.5 M NaOAc. Then 1.42 g of Na_10_[α-SiW_9_O_34_]·H_2_O was added slowly in small portions. The solution changed its color from orange to dark brown. The solution was vigorously stirred for 24 h followed by the addition of a 50% excess of KCl to precipitate K_10_[SiFe^II^_3_(H_2_O)_3_W_9_O_37_].

### Electrochemistry

Cyclic voltammetry (CV) measurements were carried out with *iR* compensation, and calibrations of potentials in THF and PEG-400 were also carried out, Figures S4 and S9. Typically, measurements were done under a He inert atmosphere of He or purified N_2_ with a dry solvent containing 0.1 M TBAPF_6_ as electrolyte and 0.5 mM TBA{SiFe_3_W_9_} in 10 mL of THF. Working Electrode – glassy carbon; counter electrode – platinum wire; and reference electrode – Fc/Fc^+^. Glassy carbon electrodes were polished before every experiment. Platinum wire electrodes were pretreated over a flame, and copper, nickel, and stainless electrodes were cleaned using 0.1 M H_2_SO_4_. The Fc/Fc^+^ reference electrode was prepared according to a literature protocol.^61^ Electrochemical nitrogen reduction reaction experiments were done using controlled potential electrolysis (CPE) using (a) A standard three-electrode undivided Pyrex cell setup, with a 1 mm diameter copper wire as working electrode, a platinum wire separated by a glass frit as a counter electrode, and Fc/Fc^+^ as a reference electrode. The solution contained 10 mL of dry solvent with 0.1 M TBAPF_6_ as electrolyte and 0.5 mM TBA{SiFe_3_W_9_}. The head space was 15 mL and “looped” with 70 mL of nonrefreshed and purified N_2_ which was continuously flowed through the cell to maximize mass transfer at the gas–liquid interface. (b) An undivided flow cell electrolyzer with two electrodes was used (no headspace) where the cathode was a 0.25 cm^2^, 0.127 mm thick copper foil (Alfa Aeasar) and the anode was a stainless-steel plate. All measurements were done with a continuous flow of 70 mL of unrefreshed gas after an initial purge of 30 min, Figure S26, using purified N_2_ that was circulated back to the system using a glass pump MR-2000N-SEB purchased from Makuhari Rikagaku Garasu Inc. The closed loop system used prevents accumulation of possible NO_*x*_ impurities from the gas feed during the reaction. Since the amounts of NH_3_ formed reached 1000+ nmol in the experiments using a copper wire in the Pyrex cell electrolyzer and up to 5100 nmol of NH_3_ in the electrolyzer with a volume of 2 mL, NH_3_ formation related to NO_*x*_ contamination is in any case negligible. For experiments using purified ^15^N_2_ and comparison of ^14^N_2_ experiments, the flow cell electrolyzer was used. All experiments were done at room temperature and 1 bar of N_2_.

### NH_3_ Quantification

The ammonia concentration was quantified by using two methods. The indophenol method:^62^ 0.1 M H_2_SO_4_ was added to the reaction sample in a 1:1 volumetric ratio. An aliquot of 1 mL was taken from the solution, which was then added to 10 mL of the color reagent consisting of 0.05 M phenol, 0.5 mM sodium nitroprusside, 0.42 mL of 6% NaClO_4_ and 0.06 M NaOH. The solution was left standing at room temperature without stirring for 1 h. Afterward the UV–vis absorption spectrum of the solution was measured. Absorbance–concentration curves were calibrated by using NH_4_PF_6_. See Figures S27 and S28. The ammonia yield was calculated as follows:

. The faradaic efficiency was calculated as follows:
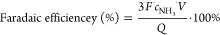
where *V* (ml) is the solution volume, *m* (mg) is the mass of the catalyst, *t* (h) is the reaction time, *c*_NH_3__ is the measured ammonia concentration, *F* (96500 C mol^–1^) is the Faraday constant, and *Q* (C) is the actual current used during the CPE in coulomb units. The NMR method:^63^^1^H NMR was measured for reactions using ^15^N_2_ and ^14^N_2_ and to verify the indophenol analyses. After CPE the reaction solution was treated with 0.1 M H_2_SO_4_ to a 1:1 volumetric ratio, and 1% DMSO-*d*_6_ was added as locking solvent. ^1^H NMR spectra at 500.08 MHz were measured using a frequency-selective pulsed gradient spin echo that is composed of a hard 90° pulse followed by two gradient pulses encompassing a selective Gaussian shaped 180° pulse as was previously reported. Calibration curves for quantitative analysis were prepared by using NH_4_PF_6_ and (^15^NH_4_)_2_SO_4_ as standards. This method was not effective for the reactions carried out with glyme due to the presence of an unidentified impurity (not noticeable in standard 1D NMR) with interfering chemical shifts.

### N_2_H_4_ Quantification

Hydrazine was quantified using the Watt and Chrisp method.^64^ From the H_2_SO_4_ treated solution another 1 mL was taken and was added to 5 mL solution of color reagent consisting of 300 mL of ethanol, 30 mL of HCl 37%, and 6 g 4-dimethylaminobenzaldehyde. The solution was left standing at room temperature without stirring for 30 min. Afterward the UV–vis absorption spectrum of the solution was measured, and absorbance–concentration curves were calibrated using hydrazine monohydrate.

### ^7^Li NMR

^7^Li NMR measurements were carried out using 0.2 mM LiClO_4_ in THF with and without {SiFe_3_W_9_} using a {SiFe_3_W_9_}/Li molar ratio of 1:25 and 1% DMSO-*d*_6_ as the locking solvent. The ^7^Li diffusion NMR measurements were conducted on a 9.4 T (155.592 MHz) AVANCE III NMR spectrometer (Bruker, Germany) equipped with a 50 gauss/cm Z gradient system. For both samples (with and without {SiFe_3_W_9_}) a stimulated echo sequence with bipolar pulsed pair gradients (BPSTE) was used at 25 °C. The diffusion experiments were performed with smoothed square (SMSQ.10.100) gradients with a total duration (δ) of 6 ms and a diffusion time (Δ) of 100 ms. The gradients were incremented from 2% to 98% in 10 linear steps, and 8 scans were acquired for each gradient. To eliminate differences in temperature/viscosity/calibration of gradients, the ^1^H diffusion coefficient of the THF signals was measured (^1^H diffusion BPLED δ = 2 ms Δ = 30 ms) and compared to the literature value of the THF self-diffusion coefficient. The differences between the measured value and the literature value were used as a factor, which was implemented in the calculation of the reported ^7^Li diffusion coefficient for each sample. Diffusion coefficient of Li in THF without {Fe_3_W_9_}: 1.39 × 10^–5^ cm^2^ s^–1^. Diffusion coefficient of Li in THF with {Fe_3_W_9_}: 0.96 × 10^–5^ cm^2^ s^–1^.

### Spectroelectrochemical Measurements - in Situ UV–vis Spectroscopy

Sample preparation was carried out in a glovebox. The samples were reduced in a quartz cuvette l = 1 cm using Pt gauze as a working electrode, Pt wire as a counter electrode, and Ag wire as a reference electrode. Solutions (5 mL) containing 4 μM TBA{SiFe_3_W_9_}, 0.01 M TBAPF_6_, and 200 μM LiClO_4_ were reduced at different potentials versus Ag as reference, and the UV–vis spectra were measured simultaneously. Reference spectra were measured with all the components except TBA{SiFe_3_W_9_} and stored within the computer of the spectrometer.

### Magnetic Susceptibility

The magnetic susceptibility of K_10_[SiFe^II^_3_(H_2_O)_3_W_9_O_37_] and K_7_[SiFe^III^_3_(H_2_O)_3_W_9_O_37_] was measured in solution using the Evans method.^52^ 0.8 mg of K_7_SiW_9_Fe_3_O_37_ or 0.7 mg of K_10_SiW_9_Fe_3_O_37_ was dissolved in a solution of 2 mL of D_2_O with 1% *tert*-butanol that was transferred into the outer part of a coaxial NMR tube, and another solution of D_2_O and 1% tert-butanol was inserted to the inner part of a coaxial NMR tube. A standard 1H pulse sequence was used in 400 MHz NMR at room temperature; the distance between the two methyl peaks caused by the paramagnetic reagent was measured. The magnetic susceptibility was calculated using the following equation: , where χ_0_ is the diamagnetic susceptibility of D_2_O = 0.72 × 10^–6^, Δ*f* is the difference in hertz between the 2 *tert*-butanol peaks, *f* is the NMR magnet strength in hertz, and *m* is the molecular weight of the polyoxometalates ∼2800. χ_g_ is converted to χ_m_ by dividing χg by 2800. Then the actual magnetic susceptibility, χ′_A_, is χ′_A_ = *χ*_*m*_ + ∑.

### Diamagnetic Corrections

The magnetic susceptibility of the diamagnetic part [SiW_9_O_34_]^10–^ is 6.22 × 10^–4^. Using the following relation, we can determine *n*, the number of unpaired electrons: , where *T* = 298 K.

For K_7_ [SiFe^III^_3_(H_2_O)_3_W_9_O_37_], *n* ≈ 15. For K_10_ [SiFe^II^_3_(H_2_O)_3_W_9_O_37_], *n* ≈ 12.

### Molecular Dynamics Simulations

Atomistic molecular dynamics (MD) simulations with explicit solvent molecules were performed to determine the cation distribution around the {SiFe_3_W_9_}^q–^ anion in solution using the GROMACS 5.1.2 code,^65,66^ and a modified AMBER 99 Force Field,^67^ which has been satisfactorily employed to study the aggregation of polyoxometalates in different environments.^68^ The force field provides the potential energy of the system as the sum of bond, angle, and dihedral deformation energies and nonbonding terms. The latter consists of pairwise additive 1–6–12 electrostatic and van der Waals potentials that account for interactions between atoms that are separated by more than three bonds. Polyoxometalate force-field parameters were obtained following the procedure by Bonet-Ávalos et al.^69^ CHelpG atomic charges derived from the electrostatic potential obtained with the Gaussian 16 package at the same level of density functional theory were used.^70^ For the MD trajectories, cubic boxes of ca. 11.2 nm edges were calculated under 3D-periodic boundary conditions, containing one {SiFe_3_W_9_} anion, 107 tetrabutyl ammonium cations, 100 PF_6_ anions, and embedding THF for 0.1 mM {Fe_3_W_9_}^q–^ anion. In the presence of a lithium salt, 400 Li^+^ cations and 400 ClO_4_^–^ anions were included in the previous box. THF molecules were described by the full-atom model provided by van der Spoel et al.^71^ For 1–4 van der Waals interactions, we applied an atom cutoff of 14 Å, and for Coulombic interactions, 14 Å was corrected for long-range electrostatics by using the particle–particle mesh Ewald (PME) summation method. All bonds were restrained by the LINCS algorithm. Production trajectories were performed within a canonical (NVT) ensemble for 20 ns, collecting data from the trajectories every 1 ps. Simulations were carried out at 298 K, and the temperature was controlled by coupling the system to a thermal bath using the velocity-rescaling algorithm. Before production runs, all systems were equilibrated by an initial 500 ps run at constant NPT to readjust the box size and a final 500 ps run at constant NVT with a relaxed solute.

### Density Functional Theory Calculations

DFT calculations were performed by employing the Gaussian16 A.03 program package.^70^ All structures were fully optimized by using the hybrid B3LYP exchange-correlation functional. The LanL2DZ(f) effective core potential basis set was applied for Fe and W while the 6-31+G* Pople basis set was used for the remaining atoms. Relativistic effects were introduced through the pseudopotentials at the W and Fe atoms.^72^ Solvent effects were included via the implicit continuum solvent model IEF-PCM with ε = 7.4257 for THF. All calculations were performed by considering high spin states for each Fe atom, as found experimentally. All attempts of forcing antiferromagnetic coupling between Fe centers were unsuccessful as standard wave function convergence criteria were never met; not even when using quadratically convergent SCF procedures (xqc keyword in Gaussian software). It has been verified in model systems containing one Fe center that the high spin-state is the most stable by more than 1 eV. Hence, the total spin is 15/2 for {SiFe^III^_3_W_9_},^69^ and 6 for {SiFe^II^_3_W_9_}. The coordination of N_2_ was studied by using the ωB97X-D exchange-correlation functional, which includes long-range and empirical dispersion corrections.

## Supporting Information Available

NMR, IR and mass spectra, magnetic susceptibility measurements, calibration curves for potentials in THF and polyethylene glycol, current versus time profiles, and additional computational results (PDF)

## Abbreviations

ATP: Adenosine Triphosphate
CPE: Controlled Potential Electrolysis
CV: Cyclic Voltammetry
DFT: Density Functional Theory
e-N2RR: Electrochemical Nitrogen Reduction Reaction
Fc/Fc^+^: Ferrocene/Ferrocenium Cation
H–B: Haber–Bosch
ICP-MS: Inductively Coupled Plasma Mass Spectrometry
MD: Molecular Dynamics
MO: Molecular Orbitals
PCET: Proton Coupled Electron Transfer
RDF: Radial Distribution Function
TBA: Tetra *n-*butylammonium
THF: Tetrahydrofuran
TOF: Turnover Frequency
TON: Turnover Number
PEG-400: Polyethylene Glycol – Molecular Weight 400
