# Supplementary material for: Electrocatalytic Reduction of Dinitrogen to Ammonia with Water as Proton and Electron Donor Catalyzed by a Combination of a Tri-ironoxotungstate and an Alkali Metal Cation
Source: J Am Chem Soc. 2023 Aug 29;145(36):19912–24. doi: 10.1021/jacs.3c06167 (PMC10510311; doi:10.1021/jacs.3c06167)
Supplement: Supplementary file 1 — ja3c06167_si_001.pdf [file ja3c06167_si_001.pdf]

Electrocatalytic Reduction of Dinitrogen to Ammonia with Water as Proton and Electron Donor Catalyzed by a Combination of a Tri-ironoxotungstate and an Alkali Metal Cation

Avra Tzaguy,[1‡] Albert Masip Sanchéz,[2‡] Liat Avram,[3] Albert Solé-Daura,[2] Xavier López,[2] Josep M. Poblet[2] and Ronny Neumann[1*]

[1]Department of Molecular Chemistry and Materials Science, Weizmann Institute of Science, Rehovot, Israel 76100.

[2]Department de Química Física i Inorgànica, Universitat Rovira i Virgili, Tarragona 43007, Spain.

[3]Department of Chemical Research Support, Weizmann Institute of Science, Rehovot, Israel 76100.

Supplementary Information

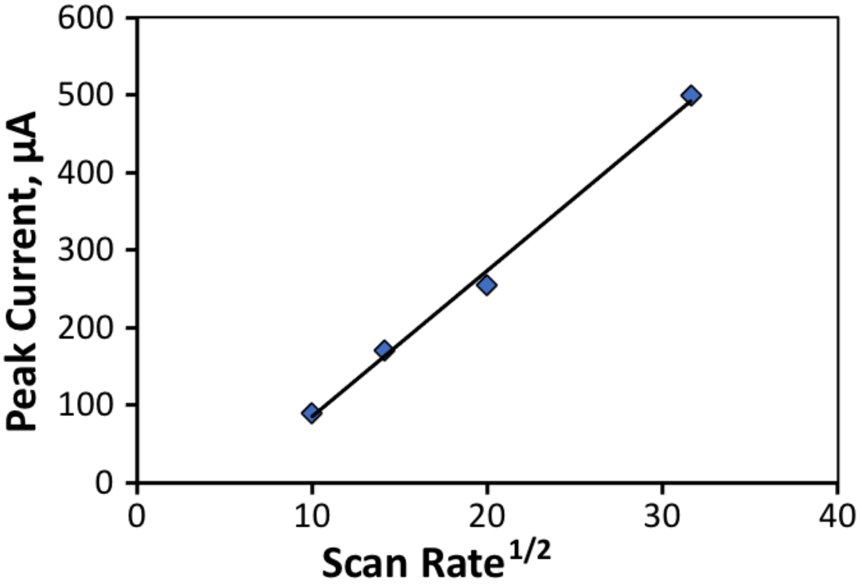

Figure S1. Randles-Sevcik plot for cyclic voltammetry measurements of TBA{$SiFe_3W_9$} under N_2 in the presence of ethanol.

Measurements were carried out in 10 mL 99:1 dry THF:ethanol containing 100 mM $TBAPF_6$ and 0.5 mM TBA{$SiFe_3W_9$}, 50 mM $LiClO_4$ under 1 bar N_2 with a glassy carbon disc working electrode, a platinum wire counter electrode, and a Fc/Fc^+ reference electrode.

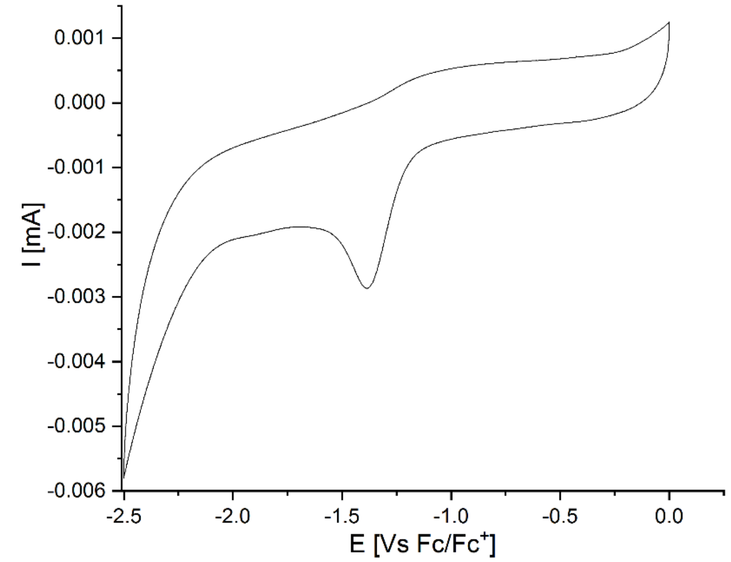

Figure S2. Cyclic Voltammetry of Aldehyde

The measurement conditions were 6 mL Dry THF containing with 0.1M $TBAPF_6$ and 110 mM aldehyde. The solution was purged for 30 min with N_2. A glassy carbon disc working electrode, a platinum wire counter electrode, and a Fc/Fc^+ reference electrode were used at a scan rate of 100mV/s

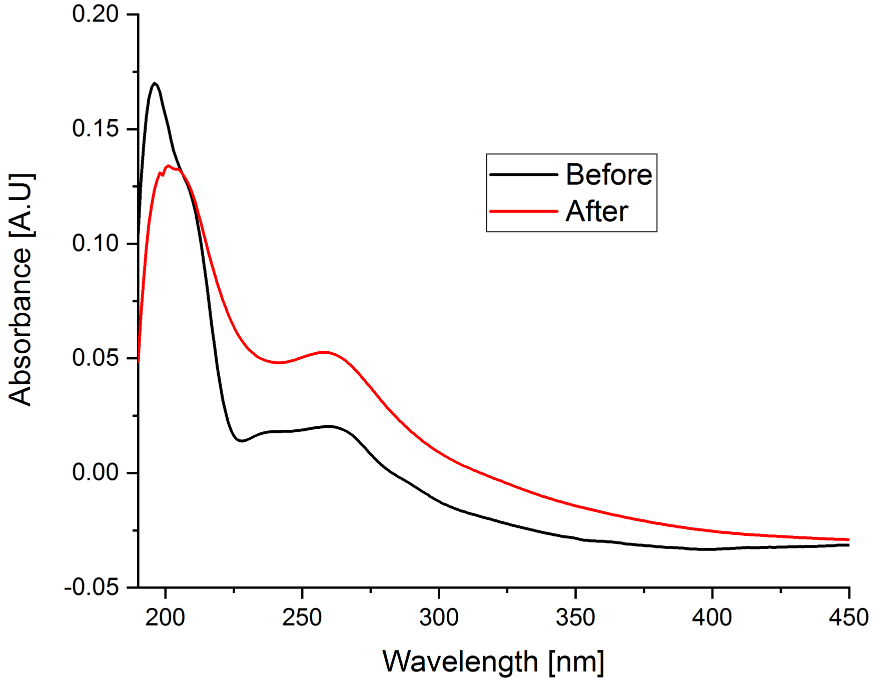

Figure S3. UV-vis spectra of TBA{$SiFe_3W_9$}, Li^+ and N_2 before and after electrolysis

There are some changes in the intensities of the peaks that in comparison with Figure 4d indicates the presence of a residual amount of 1-electron reduced species.

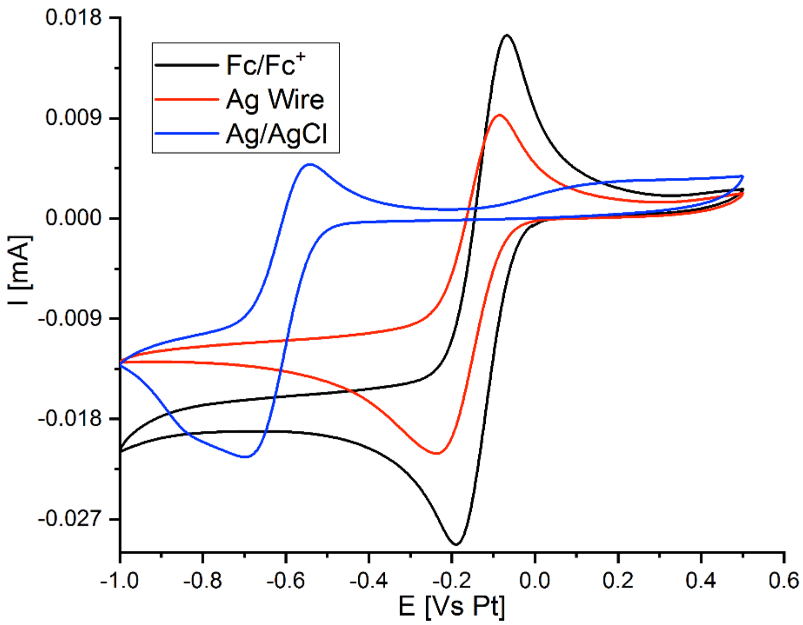

Figure S4. Calibration of Various Electrodes in dry THF in the Presence of 0.1 M $TBAPF_6$ as Electrolyte using Pt wires as Counter and Reference Electrodes.

Fc/Fc^+ was measured using a 4 mM solution of Ferrocene with a Pt disk working electrode.

Ag wire an Ag/AgCl were measured using them as working electrodes.

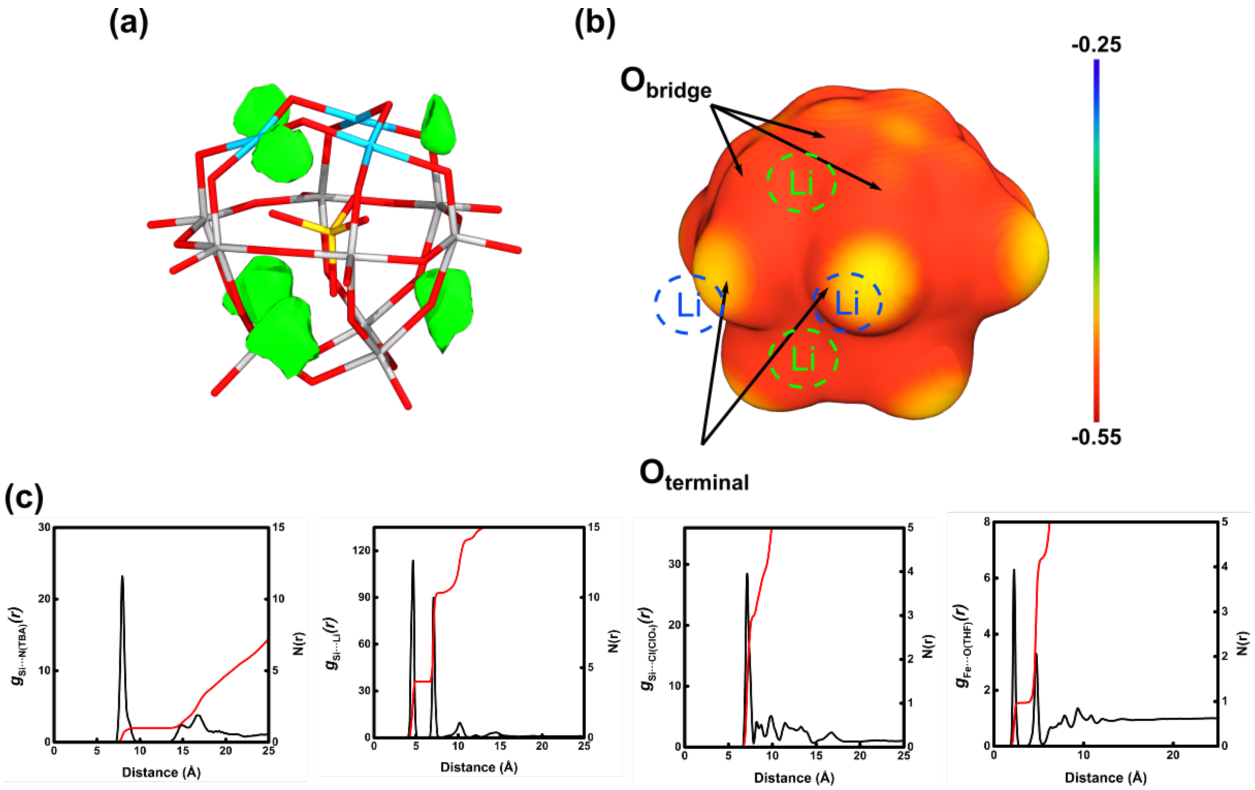

Figure S5. (a) Electrostatic isopotential surface showing the most negative potential (nucleophilic) wells of the $\{SiFe_3W_9O_{37}\}$ anion. (b) Computed molecular electrostatic potential mapped onto an isodensity surface of 0.0004 for $\{SiFe_3W_9O_{37}\}$. Lithium-center coordination modes are also included in bridge (green) and terminal (blue) oxygens. Three lithium cations preferentially approach to the three nucleophilic wells near $[Fe_3O_3]$, but only one is localized in a well generated by $[W_4O_4]$. We noticed that this Li^+ cation is also coordinated to a ClO_4^-, which is in turn attached to other Li^+ in terminal oxygens. (c) Radial distribution functions (RDFs, *g(r)*) (in black line) between the $\{SiFe_3W_9O_{37}\}$ (Si or Fe as reference) and, from left to right, N of TBA, Li^+, Cl of ClO_4^- and O of THF. Integration of the *g(r)* (in red line) is also included.

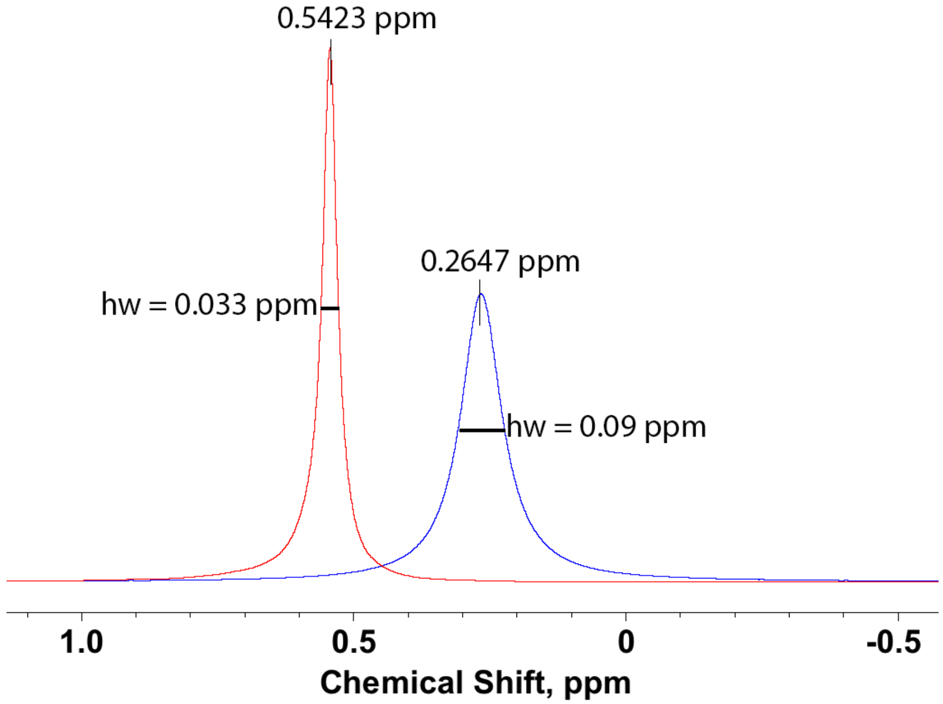

Figure S6. ^{7}Li NMR of 0.2 mM $LiClO_4$ in THF (red) and 0.2 mM $LiClO_4$ with 8 µM $\{SiFe_3W_9\}$ (blue).

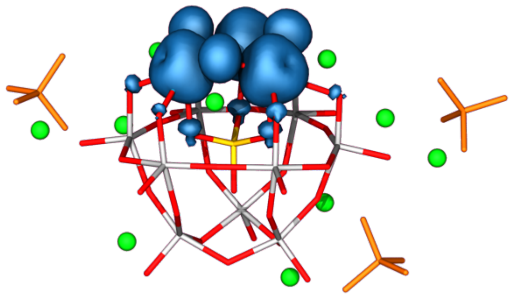

Figure S7. Spin density distribution represented in $\{SiFe^{II}{}_3W_9O_{37}\}^{10-}/10Li^+/3ClO_4^-$. Blue surfaces in the $[Fe_3O_3]$ region represent excess of ⍰-spin electron density.

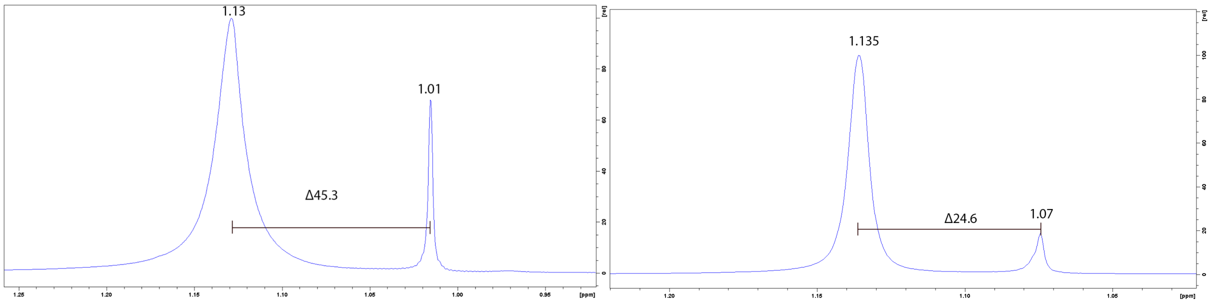

Figure S8. Magnetic susceptibility measurements using the Evans method. Left 0.8 mg $K_7[SiFe^{III}{}_3(H_2O)_3W_9O_{37}$; right 0.7 mg $K_{10}[SiFe^{II}{}_3(H_2O)_3W_9O_{37}$

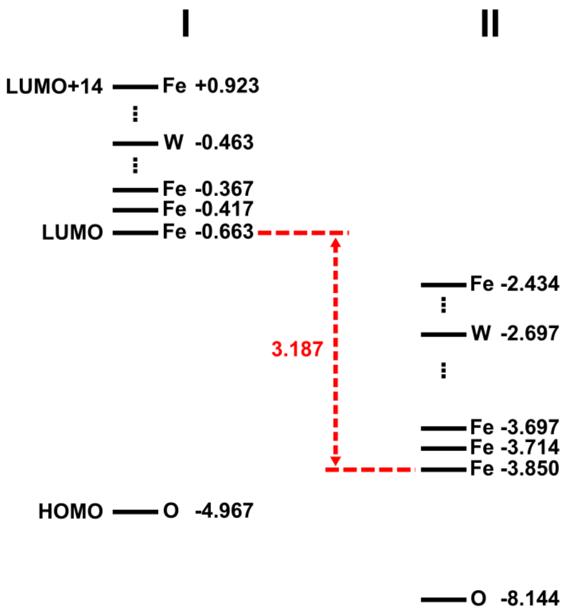

Figure S9. Schematic molecular orbital diagrams for the lowest unoccupied orbitals of the polyoxmetalate I) $\{SiFe^{III}_3W_9O_{37}\}^{7-}$ embedded in a continuum dielectric and II) $\{SiFe^{III}_3W_9O_{37}\}^{7-}$ + $10Li^+$ + $3ClO_4^-$ + continuum dielectric. Orbital energies are in eV. Fe, W and O labels represent the atoms with higher contribution to the molecular orbitals.

The reduction potential of a polyoxometalate depends on the absolute energies of the LUMOs. In the gas phase, the molecular orbitals of a polyoxometalate are, in general, very high in energy because of the negative charge of the anion. In solution, the solute polyoxometalate orbitals are much lower in energy due to the electric field created by solvent molecules and counter cations.

For highly charged compounds such as the $\{SiFe^{III}_3W_9O_{37}{}^{7-}\}$ anion under consideration here, the continuum solvent methods were unable to correctly simulate the environment (solvent + counterions) effects. The consequence is that the frontier molecular orbital energies are excessively high. In addition, and importantly, under the present experimental conditions, MD simulations show that several Li^+ ions are in direct contact with the polyoxometalate, introducing an extra stabilization of the polyoxometalate, which cannot be reproduced by an implicit solvation method. Therefore, addition of a Li^+ salt to the THF solution drastically changes the properties of the polyoxometalate anion, the redox activity being one of the most affected properties.

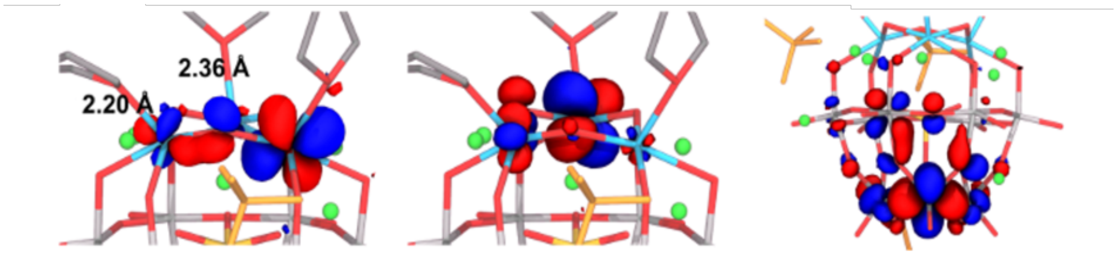

Figure S10. Representation of the three MOs occupied upon the 3e–reduction in the THF-containing model (see main text for details). Two electrons are delocalized at the three Fe centers, with a lower contribution of the Fe bound more strongly to one of the THF ligands, and the third electron is delocalized among W centers.

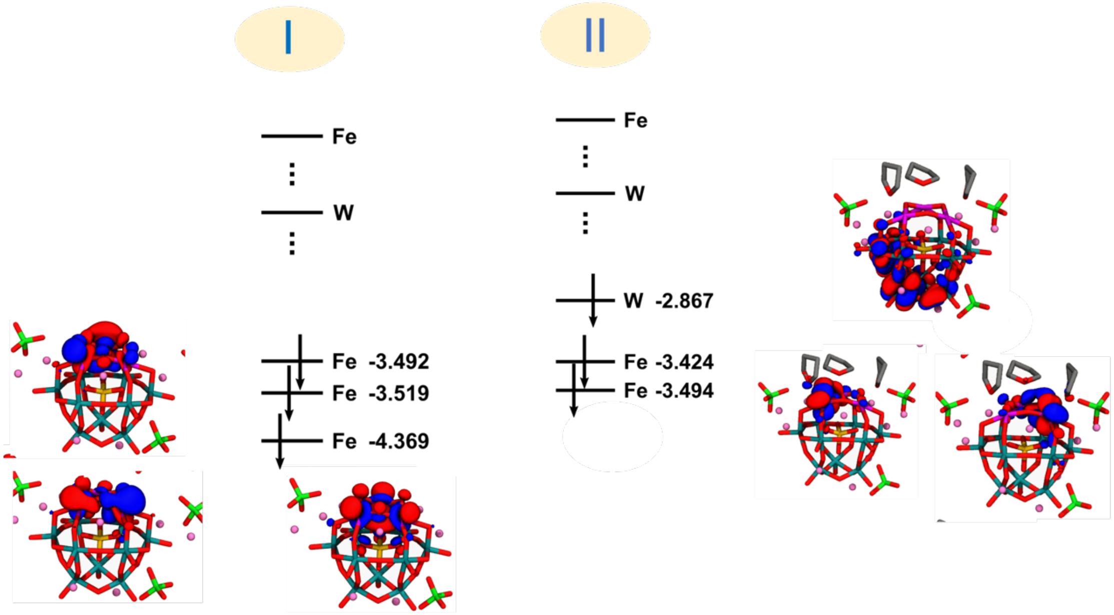

Figure S11. Schematic molecular orbital diagram for the 3-electron-reduced systems: I) $\{SiFe^{II}{}_3W_9O_{37}\}^{7-}$ + $10Li^+$ + $3ClO_4^-$ + continuum dielectric and II) $\{(THF)_3SiFe^{II}{}_2Fe^{III}W^{VI}{}_8W^{V}O_{37}\}^{7-}$ + $10Li^+$ + $3ClO_4^-$ + continuum dielectric. The binding of three solvent molecules to the polyoxometalate induces a significant change in its electronic structure. In particular, the three lowest occupied (beta) d(Fe) molecular orbitals are destabilized by the presence of THF ligands, causing the transfer of one electron to the polyoxotungstate framework. Orbital energies are in eV. Fe, W and O labels represent the atoms with higher contribution to the molecular orbital.

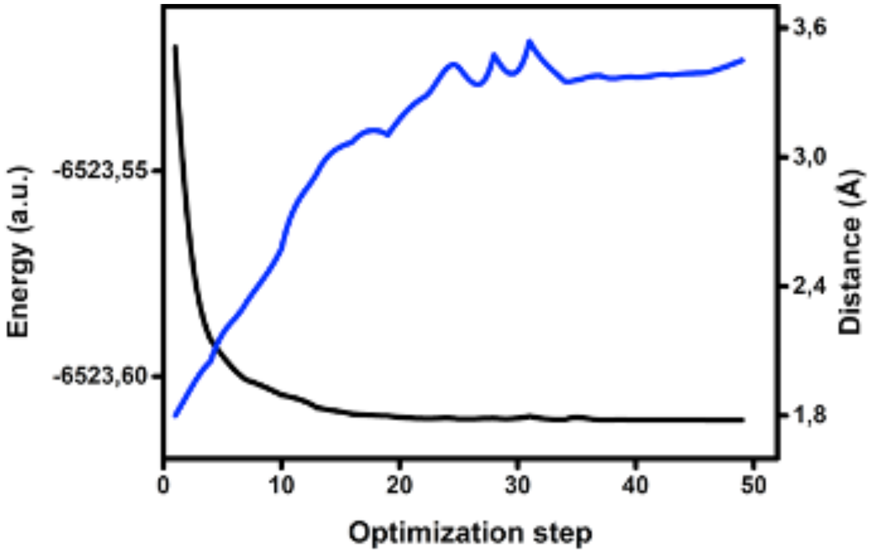

Figure S12. The curves show how the energy (blue) and distance (black) of Fe···N_2 change during the optimization process when trying to coordinate a N_2 molecule to the 3-electron reduced catalyst. In the absence of THF ligands, all attempts to bind a N_2 to one of the Fe(II) centers failed as it was impossible to find an energy minimum in the region close to 1.9 Å. We find a decrease in energy only when N_2 moves away from the metallic center.

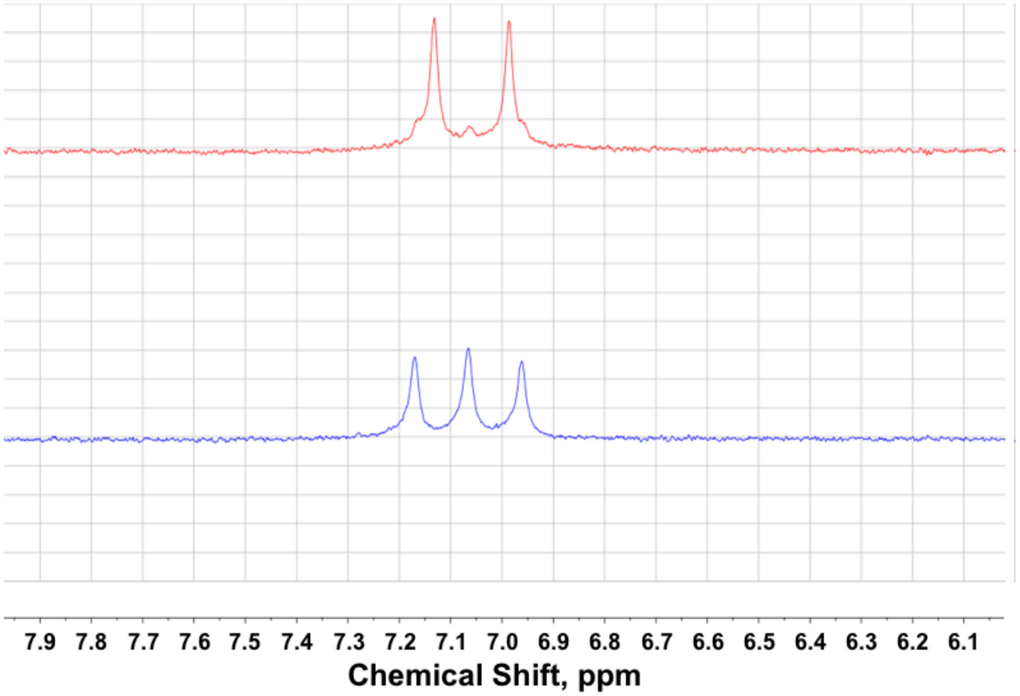

Figure S13. ^{1}H NMR (selgpse, 500.08 MHz) after 5 h CPE in an electrolyzer: 0.1 M $TBAPF_6$, 0.5 mM $\{SiFe_3W_9\}$, 25 mM $LiClO_4$ in THF with 1 vol% ethanol as proton donor under 1 bar $^{14}N_2$ (blue) or $^{15}N_2$ (red) using a copper foil as working electrode, a stainless-steel counter electrode. The residual ^{14}N peaks in the $^{15}N_2$ experiment is associated with the isotopic purity of the $^{15}N_2$ used, experimental difficulties encountered in purging $^{14}N_2$ from volatile THF, (see Figure S16 where PEG-400 was solvent and no purging difficulties were encountered) and possibly atmospheric contamination by $^{14}NH_3$. The coupling constant for $^{14}NH_3$ is 53 Hz; The coupling constant for $^{15}NH_3$ is 72 Hz.

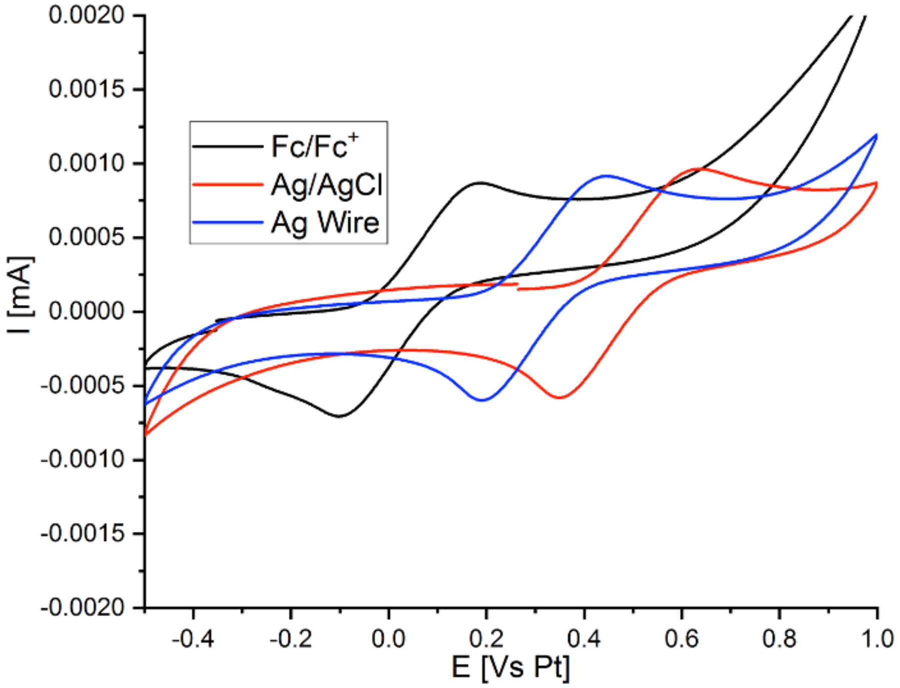

Figure S14. Calibration of Various Electrodes in dry PEG-400 in the Presence of 0.1 M $TBAPF_6$ as Electrolyte using Pt wires as Counter and Reference Electrodes.

Fc/Fc^+ was measured using a 4 mM solution of Ferrocene with a Pt disk working electrode.

Ag wire an Ag/AgCl were measured using them as working electrodes

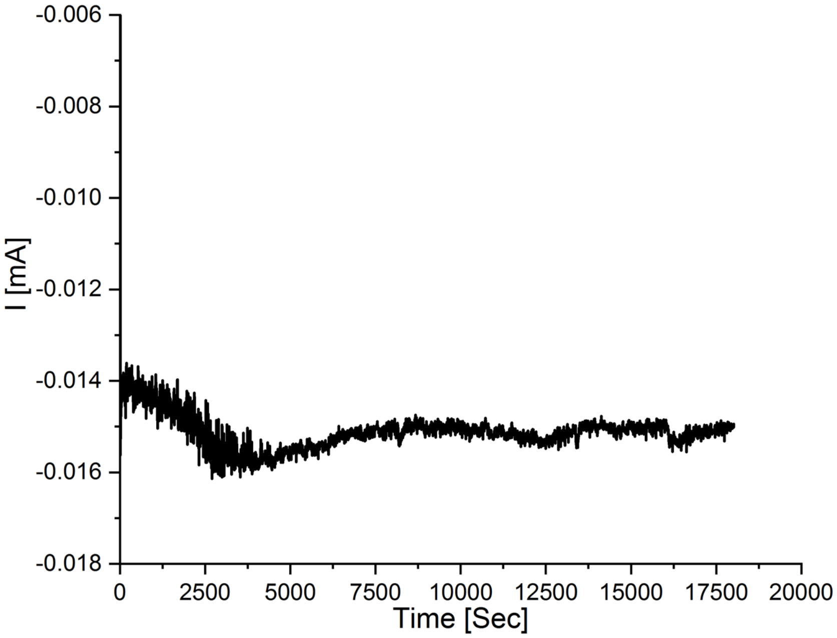

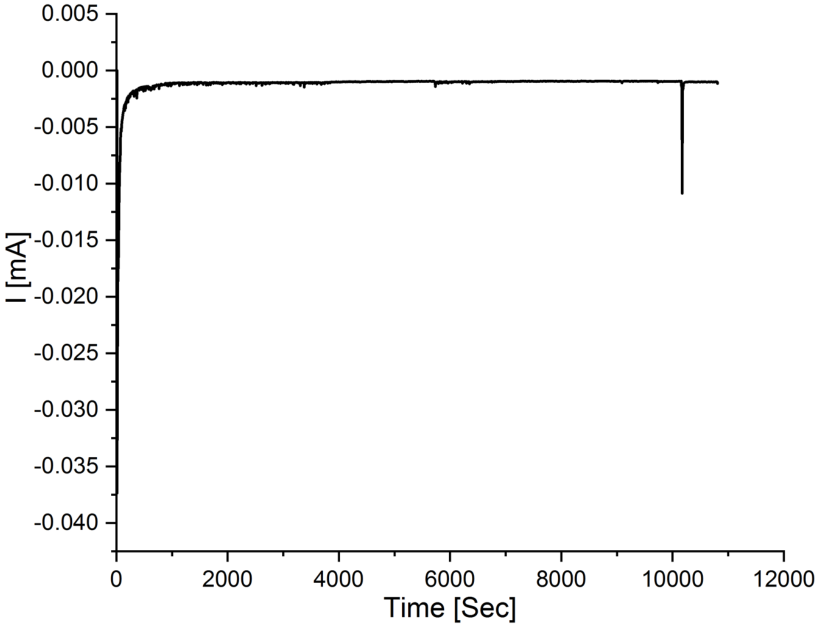

Figure S15. Current versus time plot for reaction (top) with 0.5 mM TBA{$SiFe_3W_9$} and (bottom) without TBA{$SiFe_3W_9$}.

Reaction conditions: 10 mL PEG-400 containing 0.1 M $TBAPF_6$, with or without 0.5 mM TBA{$SiFe_3W_9$}, 25 mM $NaClO_4$ with 1 vol% water under 1 bar N_2 for 3 h using a copper wire working electrode, a platinum wire counter electrode, and a Ag/AgCl reference electrode.

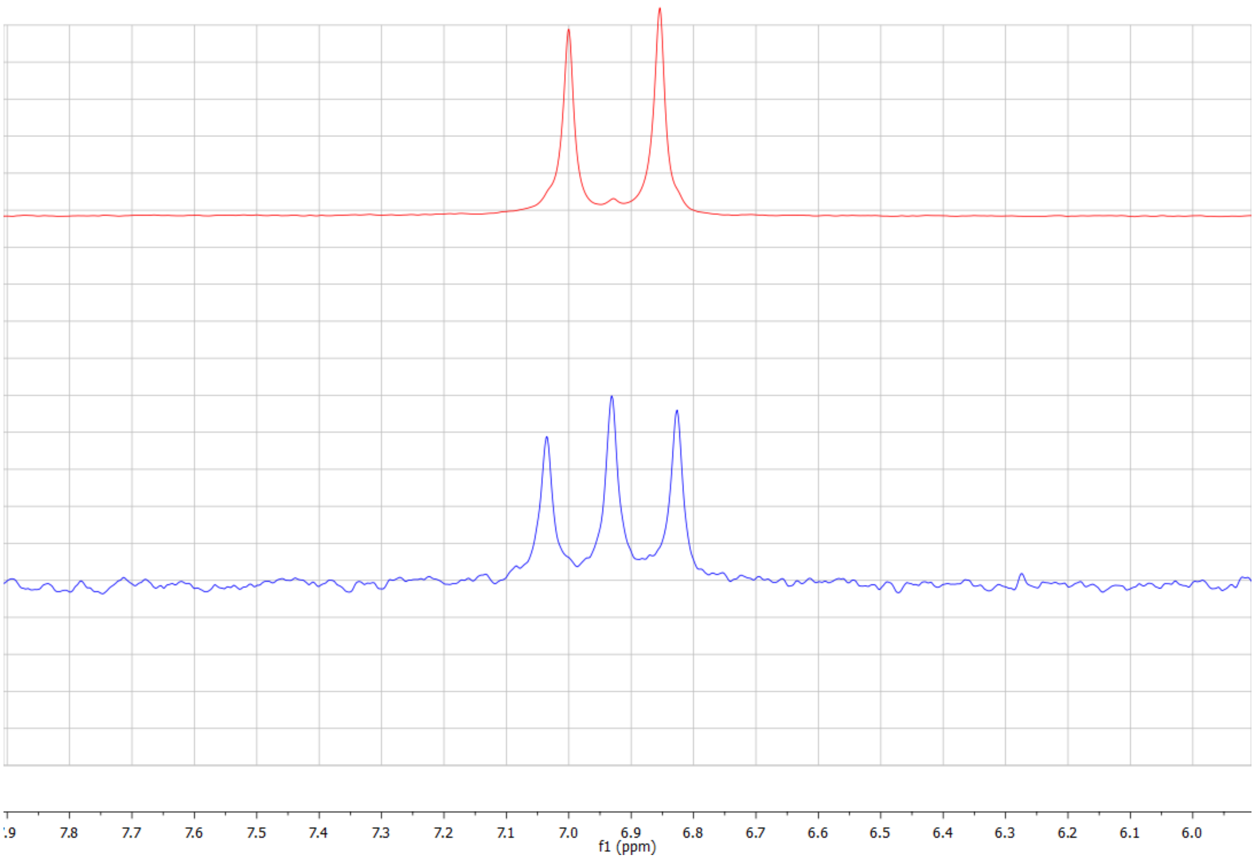

Figure S16. ^{1}H NMR (selgpse, 500.08 MHz) after 5 h CPE in an electrolyzer: 0.1 M $TBAPF_6$, 0.5 mM $\{SFe_3W_9\}$, 25 mM $NaClO_4$ in PEG-400 with 0.75 vol% water as proton donor under 1 bar $^{14}N_2$ (blue) or $^{15}N_2$ (red) using a copper foil as working electrode and a stainless-steel counter electrode.

For the $^{15}N_2$ experiment, it should be noted that due the high viscosity of PEG-400, and its low volatility, excellent results in degassing the solvent to remove $^{14}N_2$ were obtained by purging with He for 30 min at 60 °C, followed by the introduction of $^{15}N_2$. The residual $^{14}NH_3$ peak is attributed to the isotopic purity (98%) of the $^{15}N_2$ used and possibly other small contaminations. The coupling constant for $^{14}NH_3$ is 53 Hz; The coupling constant for $^{15}NH_3$ is 72 Hz.

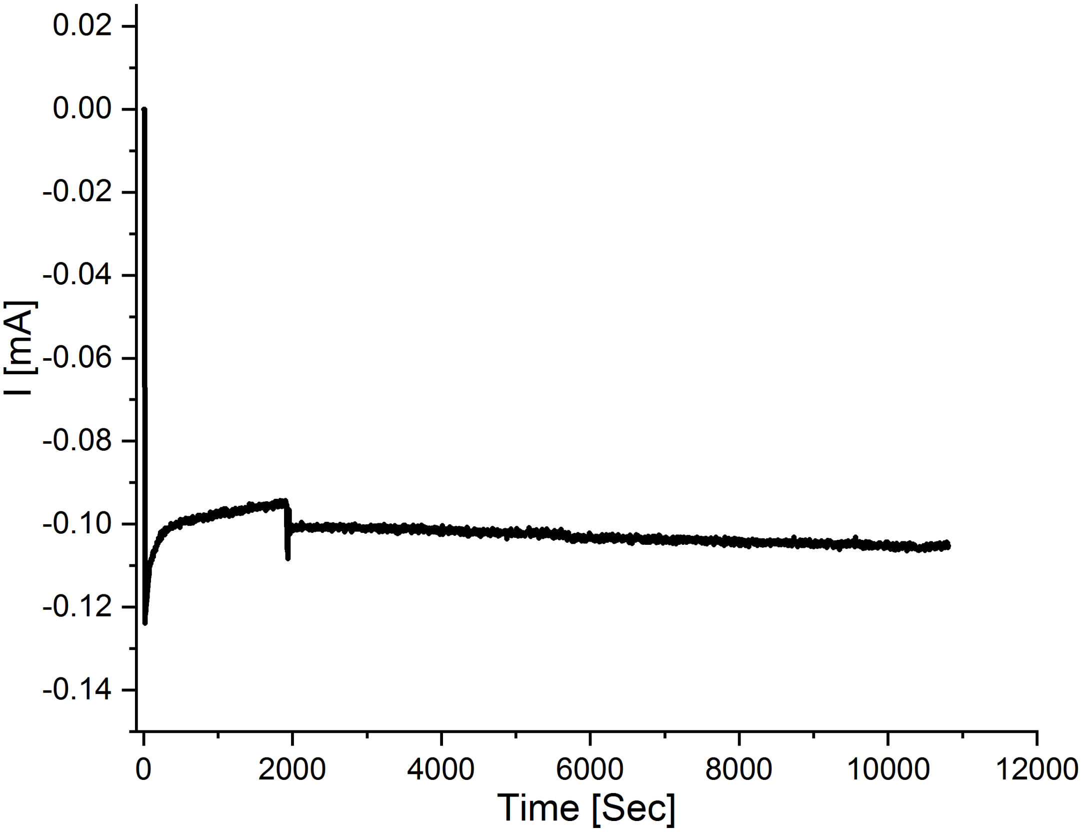

Figure S17. Current versus time profile for N_2 reduction on Cu.

The reaction was carried out in and undivided cell electrolyzer, consisting of a 0.13 cm^2 Cu foam cathode, a stainless-steel anode loaded with 2 mL PEG-400 containing 0.5 mM Na{$SiFe_3W_9$}, 1 vol% H_2O, and 0.1 M $NaCF_3SO_3$ under 1 bar N_2 operated at -1.3 V versus SHE for 3 h.

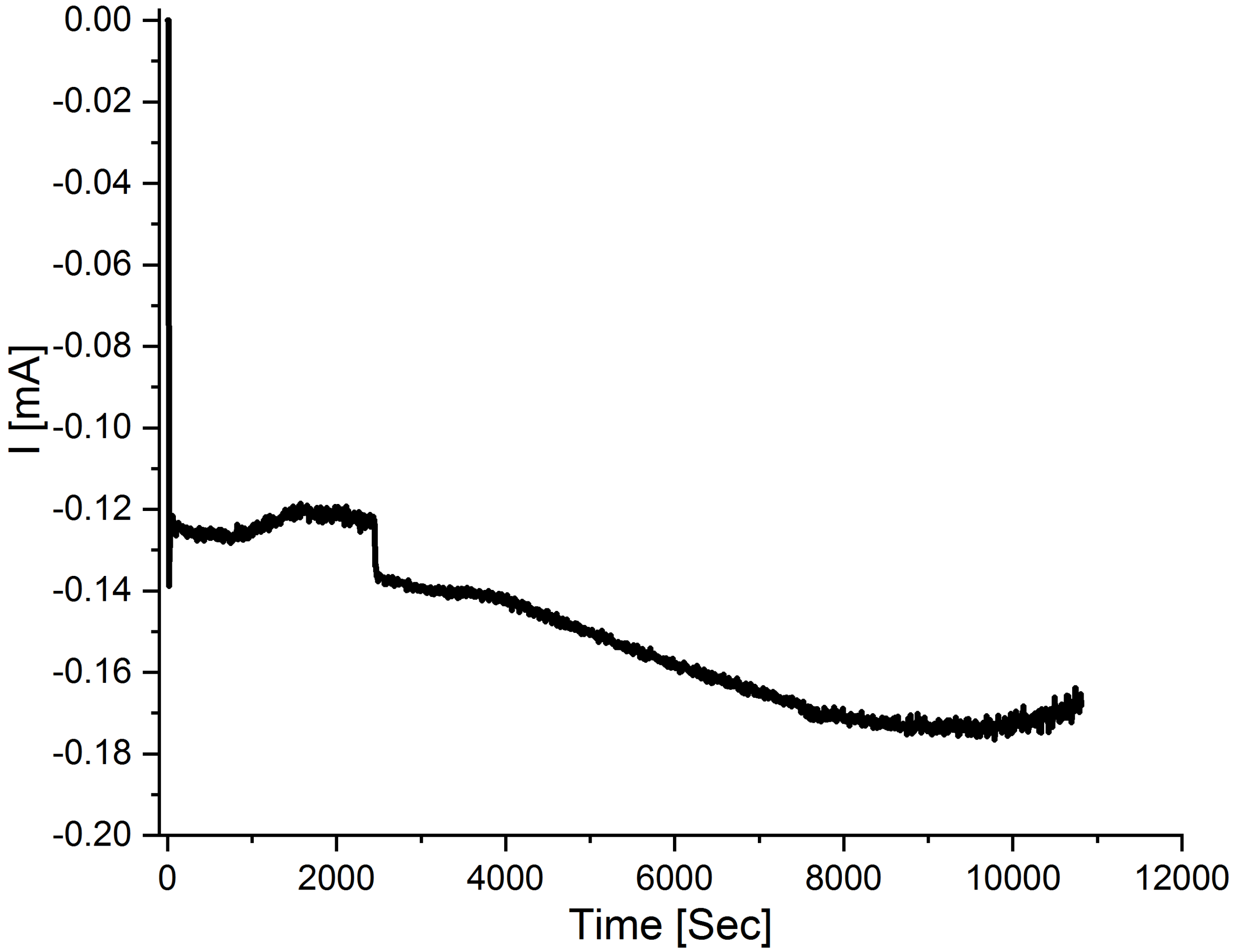

Figure S18. Current versus time profile for N_2 reduction on Ni.

The reaction was carried out in and undivided cell electrolyzer, consisting of a 0.13 cm^2 Ni mesh cathode, a stainless-steel anode loaded with 2 mL PEG-400 containing 0.5 mM Na{$SiFe_3W_9$}, 1 vol% H_2O, and 0.1 M $NaCF_3SO_3$ under 1 bar N_2 operated at -1.3 V versus SHE.

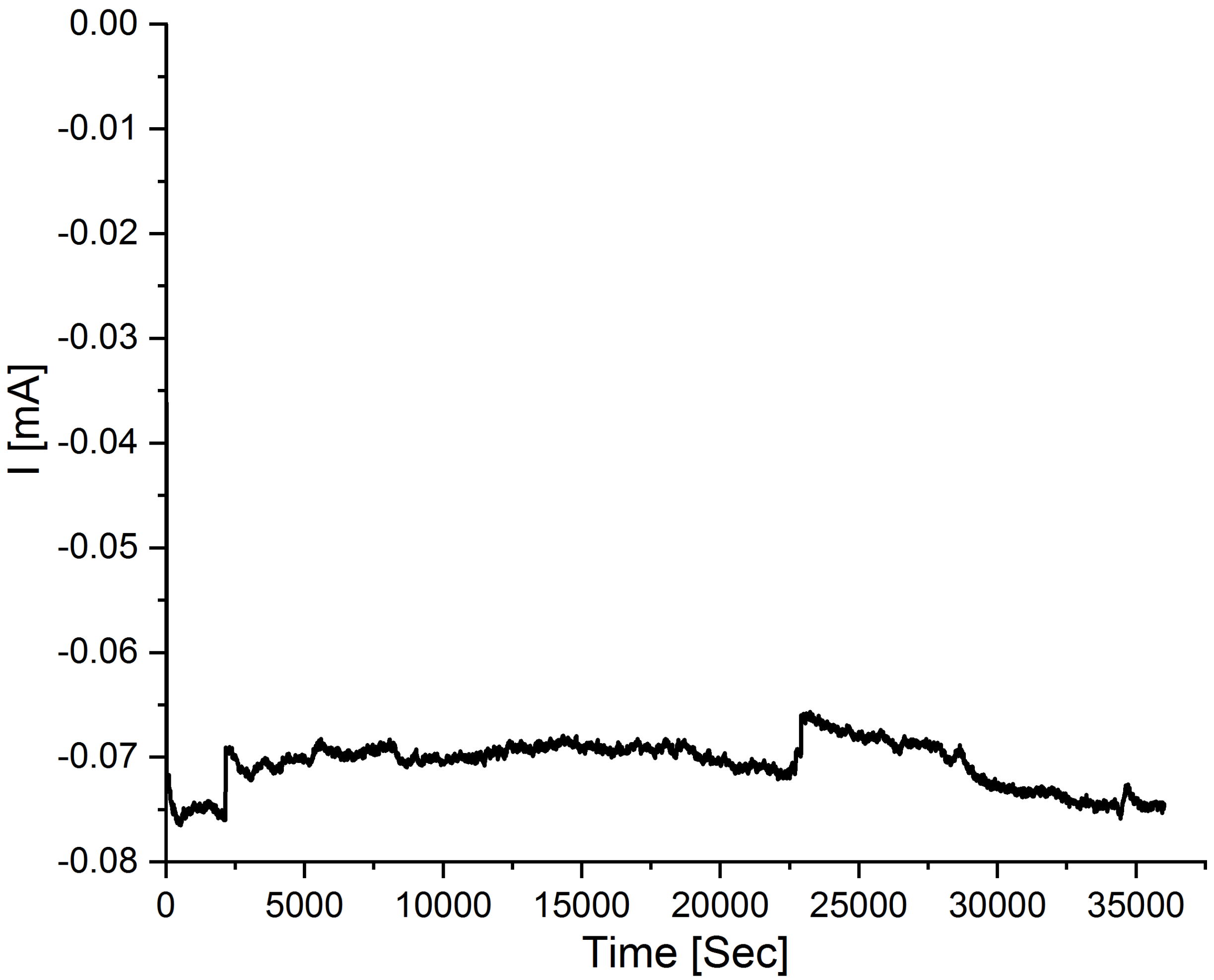

Figure S19. Current versus time profile for N_2 reduction on Cu for a 10 h reaction.

The reaction was carried out in and undivided cell electrolyzer, consisting of a 0.13 cm^2 Cu foam cathode, a stainless-steel anode loaded with 2 mL PEG-400 containing 0.5 mM Na{$SiFe_3W_9$}, 1 vol% H_2O, and 0.1 M $NaCF_3SO_3$ under 1 bar N_2 operated at -1.3 V versus SHE for 10 h.

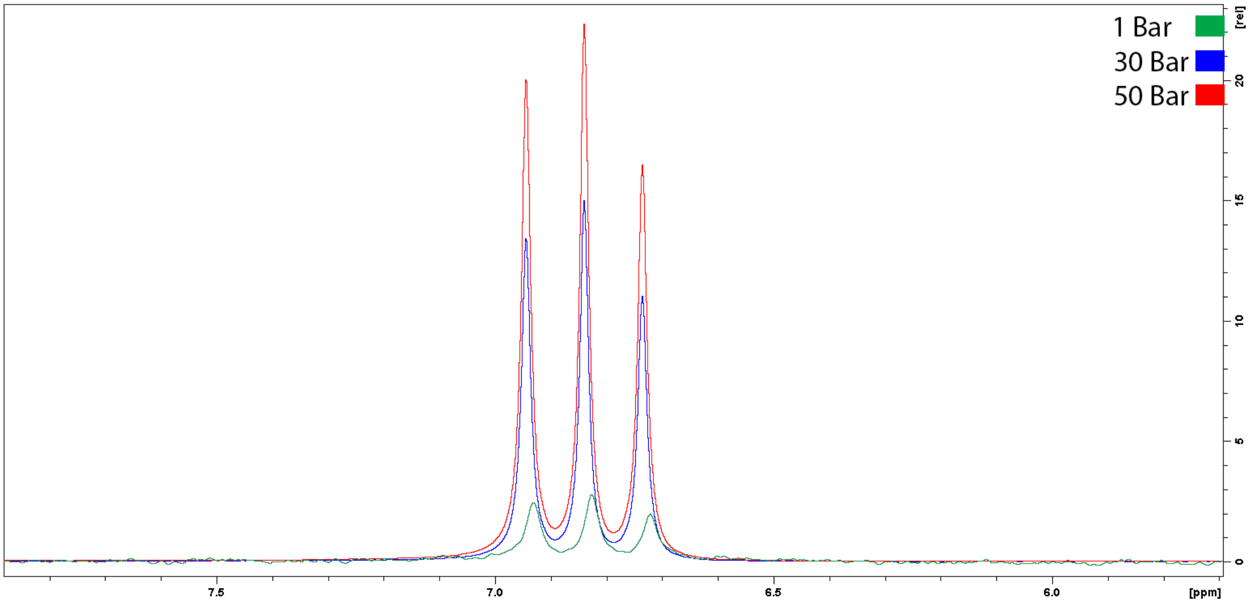

Figure S20. NMR analysis of Reactions at different N_2 Pressures. A solution of 5 ml PEG-400 containing 0.1 M $NaSO_3CF_3$as electrolyte and 0.5 mM K{$SiFe_3W_9$} with a 0.25cm^2 copper foam cathode, a stainless-steel mesh anode and a Ag/AgCl reference electrode was reacted for 1 h under 1 bar, 30 bar or 50 bar N_2 yielding 20, 115, 165 nmol NH_3, respectively.

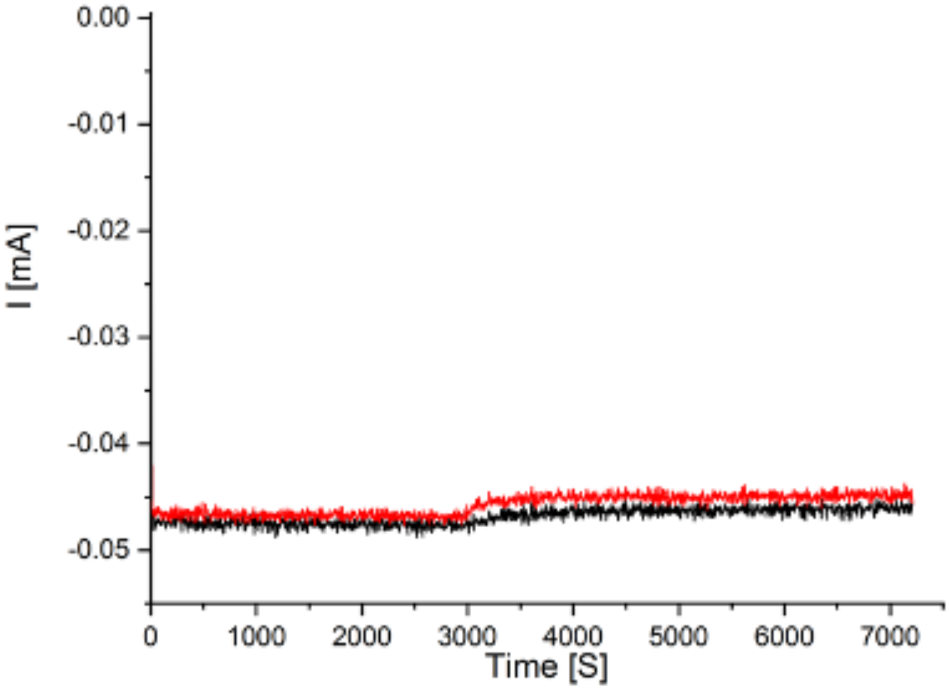

Figure S21. Recovered Cathode Experiment.

In an undivided cell electrolyzer, consisting of a 0.25 cm^2 Cu foil cathode, a stainless-steel anode loaded with 2 mL PEG-400 containing 0.5 mM Na{$SiFe_3W_9$}, 1 vol% H_2O, and 0.1 M $NaCF_3SO_3$ under 1 bar N_2 operated at -1.3 V versus SHE, the electrolyzer that yielded ~900 nmol NH_3. The current obtained is shown in black. After removal of the cathode after 2 h and a gentle wash was the reaction was continued with the same cathode for another 2 h, red line.

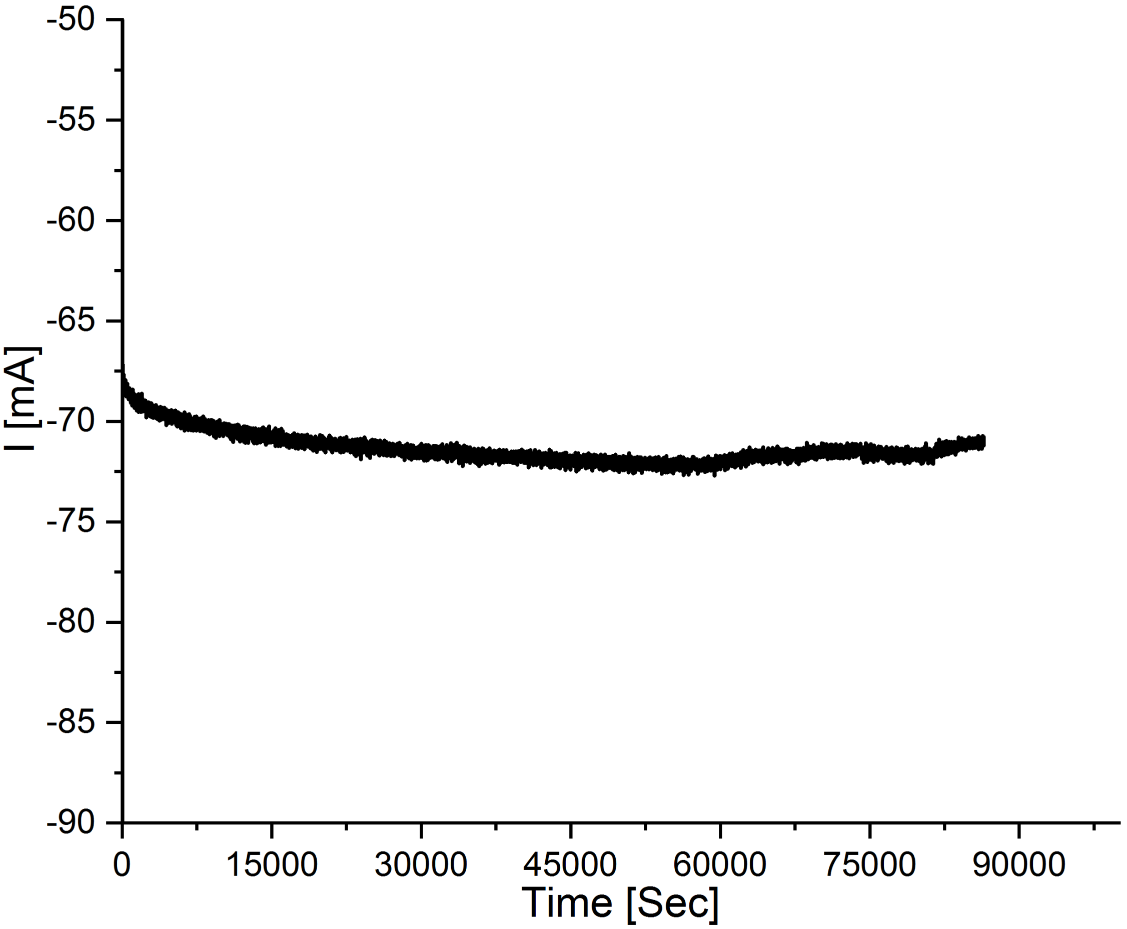

Figure S22. N_2 to NH_3 reduction in a 2 cm x 2 cm electrolyzer (divided cell).
Stainless steel cathode; titanium felt anode; zirfon perl UTP 500 membrane; 2 mL PEG-400 with 1 vol% H_2O, 0.5 mM K{$SiFe_3W_9$} and 0.1 M $NaCF_3SO_3$; t- 24 h

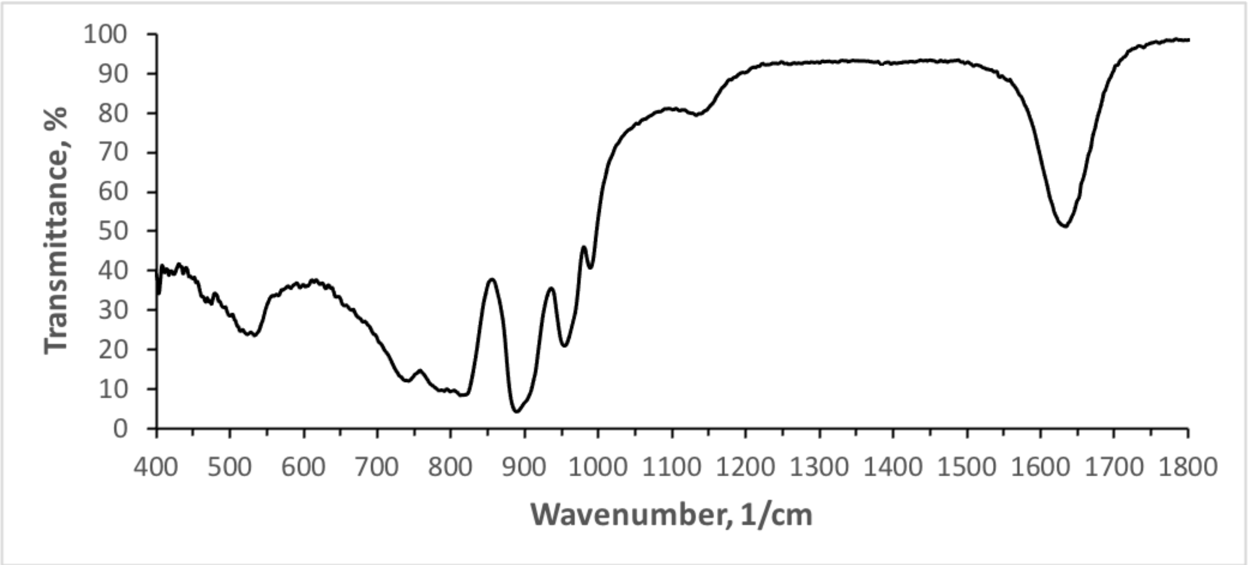

Figure S23. IR spectrum of $K_7\alpha$-[Si{Fe(H_2O)}$_3W_9O_{37}$].

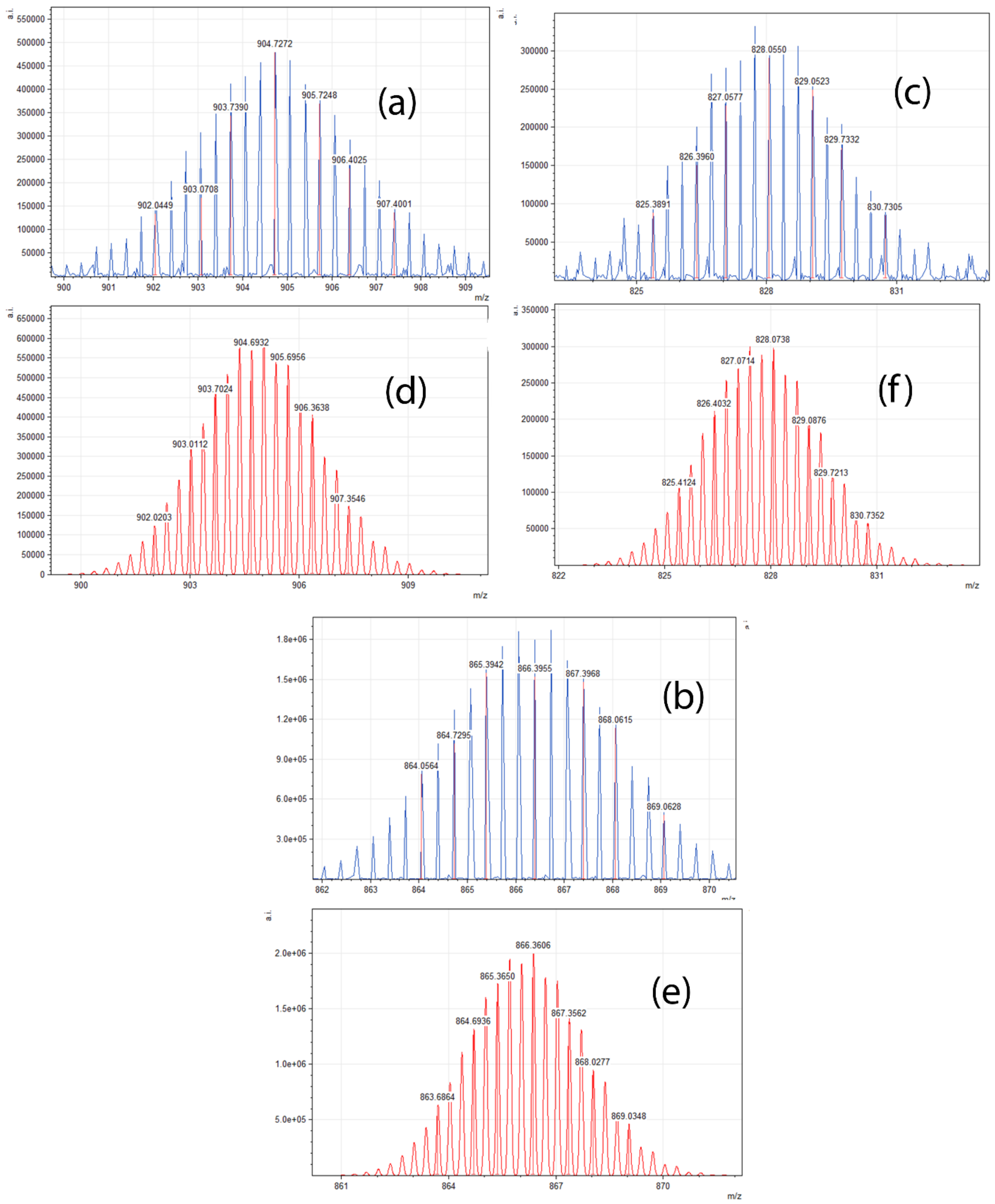

Figure S24. High resolution ESI MS of $K_7\alpha$-[Si{Fe(H_2O)}$_3W_9O_{37}$] in the presence of acetate: blue - experimental and red – simulations. a, d): K[$SiW_9O_{37}Fe_3(H_2O)(CH_3CO_2)_2$]$^{3-}$; b, e): K[$SiW_9O_{37}Fe_3(H_2O)_2(CH_3CO_2)$]$^{3-}$; c, f): K[$SiW_9O_{37}Fe_3(H_2O)_3$]$^{3-}$.

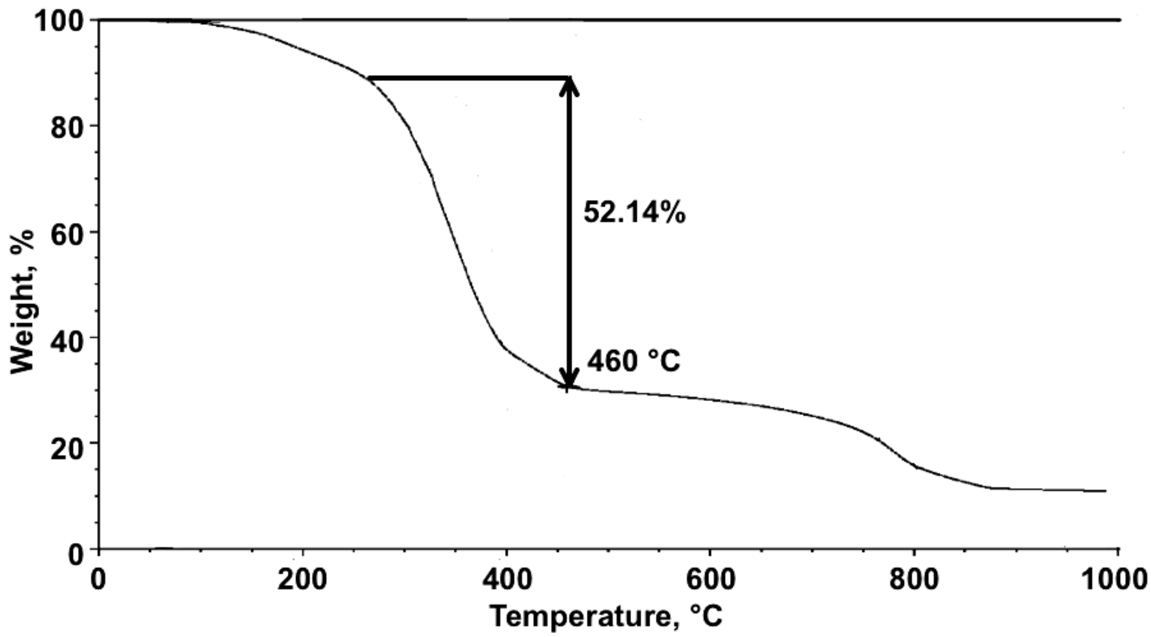

Figure S25. Thermogravimetric analysis plot of TBA{$SiFe_3W_9$}.

The weight loss between 250 and 450 °C is attributed to the pyrolysis of TBA. Thus, leading to a 10:1 ratio of ratio of TBA:α-[SiW_9O_{37}{Fe(H_2O)}$_3$] and formulation of TBA{$SiFe_3W_9$} as TBA_7[α-[$SiFe^{III}_3(H_2O)_3W_9O_{37}$]•3TBA. Note that the excess of TBA has no bearing on the electrochemical results since this cation is present in excess as electrolyte.

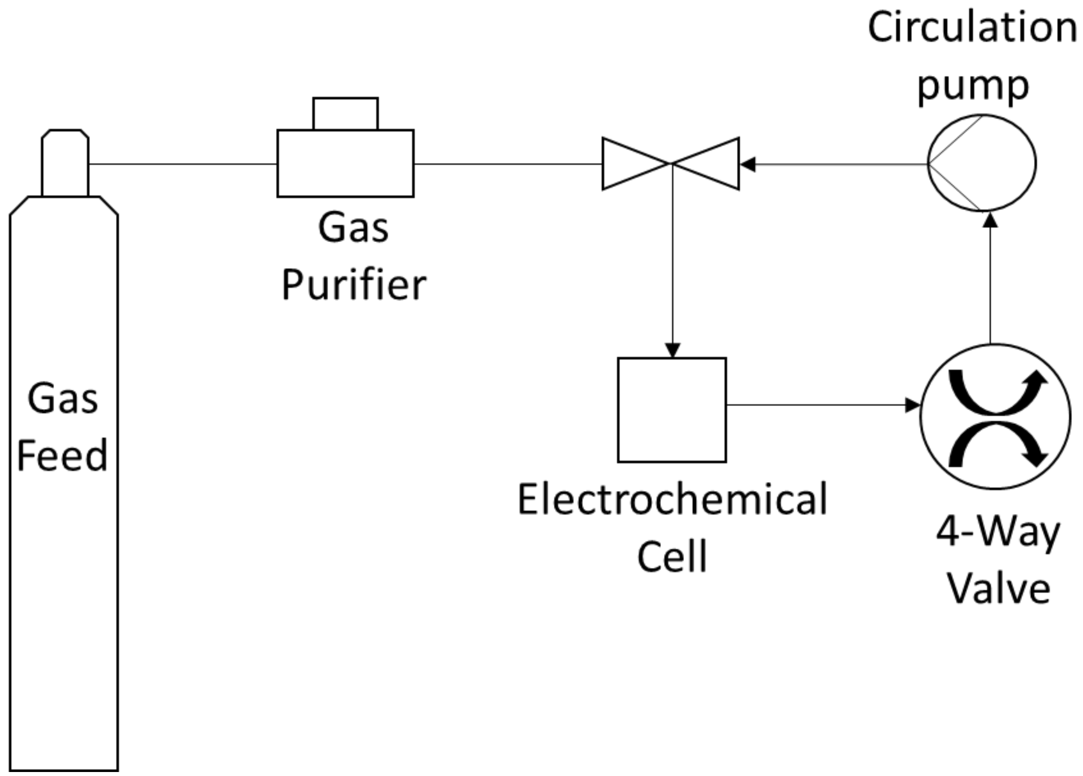

Figure S26. Electrochemical setup and gas feed circulation.

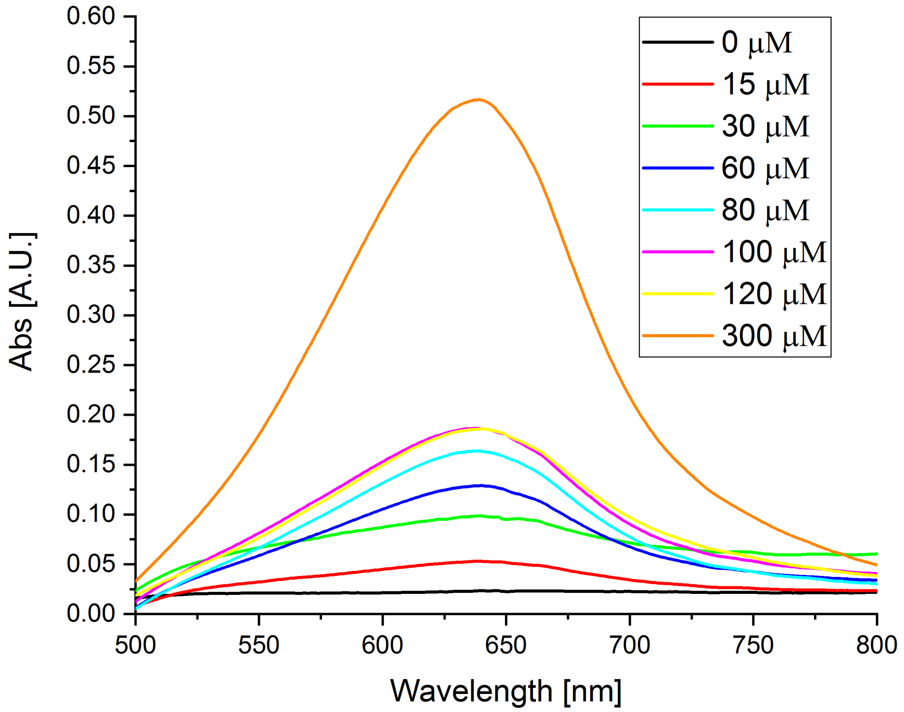

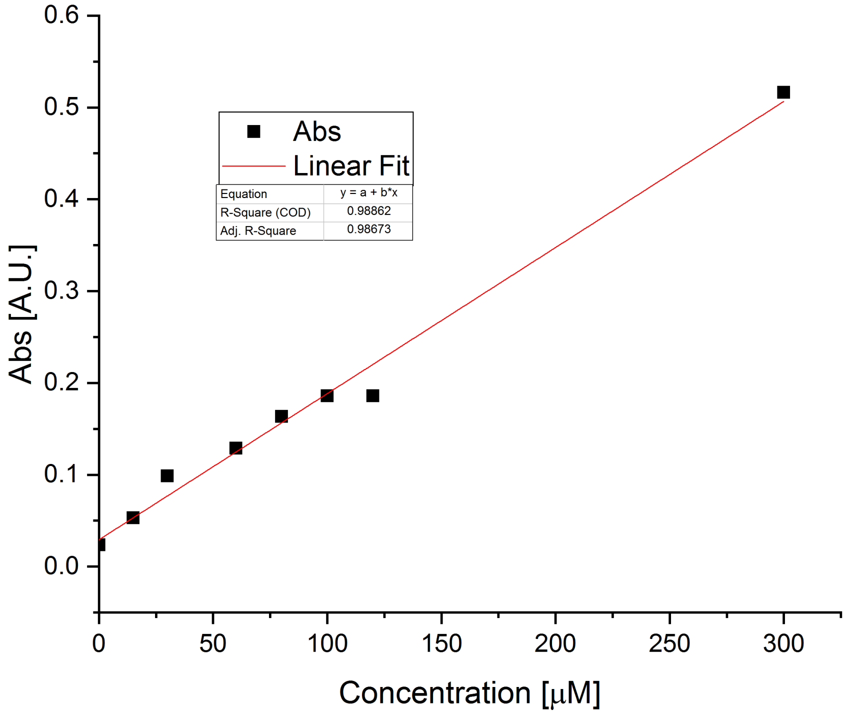

Figure S27. Visible Spectra (top) and Calibration Curve (bottom) for the Indophenol Method in THF

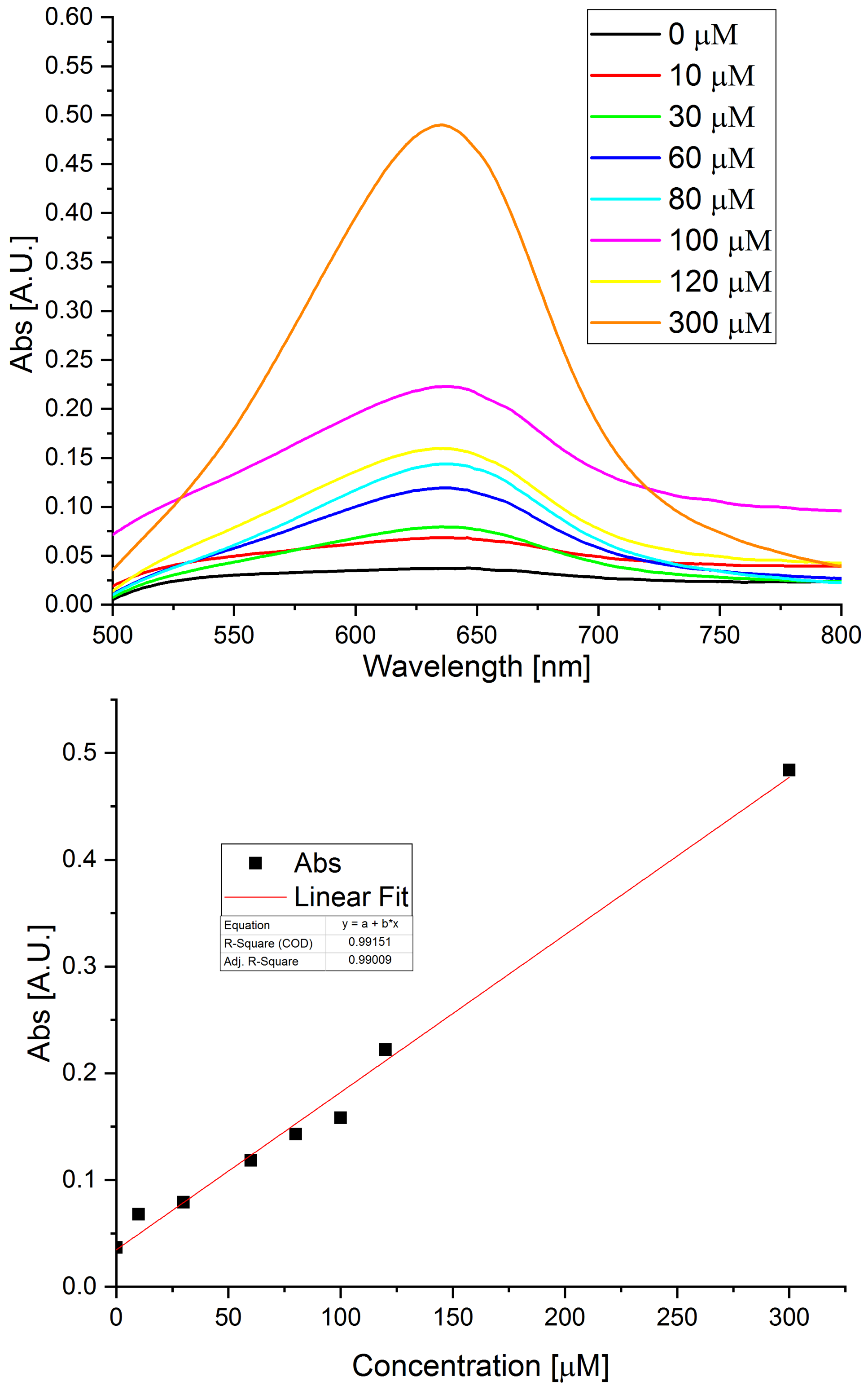

Figure S28. Visible Spectra (top) and Calibration Curve (bottom) for the Indophenol Method in PEG-400.

Table S1. Cartesian coordinates for optimized geometries for {$SiFe_3W_9$} + 10 Li^+ + 3 ClO_4^- fully oxidized and three-electron reduced.

{$SiFe_3W_9$} + 0e^- / q = 0 / -6414.93734898 a.u.				{$SiFe_3W_9$} + 3e^- / q = -3 / -6415.36123655 a.u.			
Si	0.324907	-0.306776	-0.434376	Si	0.310997	-0.455348	-0.509669
W	3.838398	0.102976	0.224324	W	3.821751	-0.121135	-0.144947
W	1.391612	3.035383	0.348517	W	1.603733	2.919228	-0.693354
W	1.62854	-1.62374	2.595156	W	1.705403	-1.042234	2.732042
W	2.741605	-2.962869	-0.654686	W	2.577645	-3.231508	-0.129847
W	-1.849937	2.539543	-0.373393	W	-1.671902	2.373696	-1.153598
W	-0.526442	0.964061	2.728074	W	-0.290946	1.635139	2.239309
W	-1.632568	-2.157525	1.870248	W	-1.638501	-1.563672	2.290249
W	-0.940689	-3.539897	-1.485526	W	-1.150458	-3.740349	-0.679689
W	-3.091639	-0.957324	-1.33557	W	-3.179198	-1.12099	-1.094153
O	3.53479	-4.493576	-0.469364	O	3.273673	-4.690788	0.555899
O	2.559026	-2.304202	3.851565	O	2.641356	-1.417788	4.127487
O	2.812521	-0.382125	1.809205	O	2.911337	-0.13244	1.638496
O	1.969263	-2.754163	1.135635	O	1.923032	-2.530878	1.658213
O	-1.052356	-3.215114	0.450477	O	-1.189191	-2.977348	1.188253
O	1.533914	3.436639	-1.401043	O	1.922796	2.987488	-2.417437
O	0.774459	-0.143669	3.536029	O	0.959487	0.683239	3.301995
O	2.843939	1.777848	0.220075	O	2.915271	1.581645	-0.378226
O	5.34133	0.61726	0.921132	O	5.384133	0.457526	0.399392
O	2.17244	4.367106	1.134553	O	2.443071	4.349676	-0.12321
O	4.08568	-1.776053	0.065462	O	4.025132	-1.980981	0.182061
O	-1.357117	2.990682	-2.059603	O	-1.361401	2.251089	-2.90757
O	1.861347	-0.752686	-0.834112	O	1.806256	-1.118817	-0.786447
O	-2.725942	0.940585	-1.033374	O	-2.726745	0.769037	-1.16732
O	-3.223651	3.554952	-0.08172	O	-2.964849	3.551062	-1.061788
O	0.044059	-0.633107	1.137993	O	0.081734	-0.325697	1.133316
O	0.785588	1.996825	1.924943	O	0.976441	2.479387	1.272925
O	0.153586	1.306148	-0.734322	O	0.248647	1.068635	-1.16842
O	-4.826128	-0.948087	-1.33783	O	-4.915635	-0.982126	-0.895628
O	-1.728663	1.567835	1.342592	O	-1.536096	1.955691	0.815065
O	-0.442404	3.60256	0.355459	O	-0.193978	3.555258	-0.820297
O	-2.728144	-1.183381	0.580062	O	-2.753076	-0.912851	0.877469
O	-1.804468	-0.499512	2.906679	O	-1.721718	0.332871	2.895163
O	-1.039324	1.924738	4.075458	O	-0.846856	2.776753	3.439731
O	-0.720183	-1.121103	-1.411387	O	-0.862938	-1.413753	-1.168808
O	-2.721831	-2.817095	-1.455104	O	-2.916419	-2.972352	-0.767433
O	-0.065436	-2.539743	2.863326	O	-0.036589	-1.742952	3.296709
O	-2.786609	-3.132978	2.658092	O	-2.773919	-2.249747	3.382861
O	1.000603	-3.501755	-1.26209	O	0.780768	-3.778322	-0.445845
O	-1.240147	-5.216758	-1.579035	O	-1.535679	-5.371	-0.289486
O	-2.688988	-0.735114	-3.092121	O	-3.01349	-1.265008	-2.850987
O	3.196614	-2.444569	-2.329773	O	2.991865	-3.389585	-1.839026
O	-0.744434	-3.034243	-3.227011	O	-0.962302	-3.781596	-2.455911

O	1.226949	-1.230777	-3.778504	O	1.161745	-2.504016	-3.839566
O	-0.646194	0.935192	-3.677217	O	-0.929447	-0.146275	-4.409559
O	4.145904	0.311112	-1.556347	O	4.051886	-0.24534	-1.910905
O	2.147567	1.376788	-3.040841	O	2.168625	0.52833	-3.91084
Fe	-0.606438	-0.941915	-3.474964	Fe	-0.585459	-1.774284	-3.466229
Fe	0.438365	2.06108	-2.660678	Fe	0.368474	0.976014	-3.554381
Fe	2.579426	-0.427058	-2.751948	Fe	2.285787	-1.195422	-3.049928
Li	1.369287	-3.085693	-3.403709	Li	1.181035	-3.904956	-2.700159
Li	3.531469	5.687735	1.08119	Li	4.022662	4.90155	0.661651
Li	4.516659	-5.941865	0.257708	Li	3.509664	-5.731259	2.075778
Li	-6.273707	-0.528594	-2.485252	Li	-6.59078	-0.155989	-0.900279
Li	-2.480304	1.962426	5.333882	Li	-2.226399	1.544244	4.308456
Li	3.336551	2.304514	-1.873663	Li	3.248131	1.508937	-2.859424
Li	-2.449114	1.381781	-3.160869	Li	-2.441726	0.478856	-3.643678
Li	-3.26124	0.356051	1.734528	Li	-3.030837	0.897886	1.418315
Li	6.783091	1.714232	0.353965	Li	6.843765	1.574533	0.418594
Li	-4.877431	4.13268	-0.769943	Li	-4.717353	4.128799	-0.99227
Cl	-5.871241	2.492507	-3.337981	Cl	-7.289222	2.698019	-1.94498
O	-6.353944	3.45682	-4.349732	O	-8.613394	3.078544	-2.495158
O	-6.682483	1.225825	-3.427004	O	-7.474783	1.619023	-0.898222
O	-6.063557	3.058481	-1.951237	O	-6.675998	3.903693	-1.264144
O	-4.425746	2.181523	-3.563955	O	-6.37366	2.207719	-3.013254
Cl	5.826852	4.544808	-0.961601	Cl	7.043432	4.644517	-0.115338
O	5.325554	5.233799	0.280167	O	5.98021	4.668864	0.967275
O	6.523689	5.518426	-1.827844	O	8.025236	5.729243	0.131485
O	4.678086	3.930561	-1.696761	O	6.400581	4.7865	-1.449901
O	6.798859	3.474114	-0.537826	O	7.755263	3.310974	-0.028854
Cl	-5.280679	1.454902	3.933556	Cl	-5.167161	2.12099	3.45904
O	-4.398464	1.705751	2.730188	O	-4.424165	2.194538	2.144542
O	-4.459263	1.711312	5.183	O	-4.15166	1.97836	4.576928
O	-5.749878	0.0441	3.932	O	-6.075772	0.942558	3.464269
O	-6.422991	2.405022	3.909699	O	-5.930535	3.381743	3.657865

Table S2. Cartesian coordinates for optimized geometries for {$THF_3SiFe_3W_9$} + 10 Li^+ + 3 ClO_4^- fully oxidized and three-electrons reduced.

{$THF_3SiFe_3W_9$} + 0e$^-$ / q = 0 / -7112.38101390 a.u.				{$THF_3SiFe_3W_9$} + 3e$^-$ / q = -3 / -7112.73822494 a.u.			
Si	0.159825	-0.24022	-0.32018	Si	0.042784	-0.32178	-0.276063
W	3.410874	-1.579388	0.397367	W	2.8746	-2.332246	0.275978
W	1.189509	-0.041641	3.097544	W	1.22562	-0.340529	3.110783
W	0.594782	-3.788627	-0.416328	W	-0.364997	-3.914494	-0.407608
W	2.364968	-1.603051	-2.793523	W	1.81904	-2.099988	-2.855906
W	-1.805676	1.215045	2.296579	W	-1.367608	1.52859	2.432164
W	-1.364336	-2.461453	1.973664	W	-1.809106	-2.151599	2.063918
W	-2.414194	-2.521812	-1.282342	W	-3.024399	-1.937295	-1.15902
W	-1.026526	-0.136436	-3.74089	W	-1.168791	0.128889	-3.661038
W	-2.98192	1.150031	-1.3452	W	-2.680249	1.77171	-1.192448
O	2.983656	-2.535732	-4.128323	O	1.954823	-3.244297	-4.178597
O	1.154099	-5.396476	-0.572852	O	-0.22407	-5.616432	-0.57264
O	2.000025	-2.939631	0.491854	O	1.31404	-3.380332	0.411161
O	1.207955	-2.971583	-1.991541	O	0.374097	-3.25678	-2.066542
O	-1.587973	-1.764365	-2.761061	O	-2.149793	-1.323033	-2.664569
O	1.794993	1.633859	2.885572	O	2.187288	1.139262	2.9291
O	-0.371101	-3.90893	1.271399	O	-1.251466	-3.775092	1.347359
O	2.547499	-0.742595	1.933634	O	2.403247	-1.388915	1.959612
O	4.698172	-2.443102	1.188931	O	4.112749	-3.384925	0.911088
O	1.837973	-0.49483	4.646627	O	1.886538	-1.056093	4.560742
O	3.584884	-2.17274	-1.399342	O	2.975274	-2.910511	-1.564168
O	-0.93716	2.781312	2.117929	O	-0.193706	2.857508	2.199391
O	1.721486	-0.435801	-0.834252	O	1.476626	-0.905904	-0.862517
O	-2.591638	1.442867	0.546696	O	-2.293463	2.019083	0.748263
O	-3.165744	1.637164	3.298232	O	-2.626755	2.283813	3.379285
O	-0.52047	-1.719804	-0.07078	O	-0.968387	-1.62432	0.006455
O	0.157342	-1.702904	2.697777	O	-0.172231	-1.763902	2.820211
O	0.155778	0.623685	1.091661	O	0.300318	0.464897	1.163069
O	-4.677887	1.544335	-1.357595	O	-4.182606	2.664207	-1.227206
O	-2.174254	-0.717409	2.014297	O	-2.203384	-0.245286	2.146525
O	-0.554081	0.593409	3.599227	O	-0.298011	0.659986	3.739815
O	-3.107747	-0.75628	-0.867291	O	-3.290979	-0.018982	-0.706098
O	-2.736403	-2.835507	0.63169	O	-3.410913	-2.12448	0.806789
O	-2.135795	-3.220998	3.333003	O	-2.825955	-2.755952	3.342888
O	-0.681184	0.601618	-1.464927	O	-0.660444	0.701324	-1.360733
O	-2.724116	0.52253	-3.121371	O	-2.656238	1.190572	-2.980801
O	-1.160106	-3.944859	-1.234593	O	-2.180131	-3.594262	-1.16383
O	-3.780434	-3.303699	-1.944063	O	-4.576972	-2.362133	-1.755312
O	0.796552	-0.82699	-3.568133	O	0.395057	-0.96378	-3.530591
O	-1.400911	-0.590132	-5.347452	O	-1.739812	-0.260513	-5.239585
O	-2.196104	2.728199	-1.726264	O	-1.615431	3.223768	-1.528903
O	3.244798	-0.035031	-2.9437	O	3.014516	-0.842669	-3.243239
O	-0.375799	1.540861	-3.915078	O	-0.195296	1.604155	-3.932862

O	1.775649	2.221701	-2.37818	O	2.314856	1.851741	-2.575419
O	0.069047	3.370143	-0.331592	O	0.929697	3.441436	-0.227354
O	4.182158	0.004187	0.005287	O	4.025304	-0.921711	-0.088347
O	2.677039	2.250628	0.369918	O	3.278335	1.756127	0.45426
Fe	-0.042886	2.597654	-2.054534	Fe	0.641759	2.641066	-2.069701
Fe	0.998814	2.660991	1.132122	Fe	1.766353	2.535583	1.107705
Fe	2.969143	1.315317	-1.236658	Fe	3.242453	0.755734	-1.323989
Li	1.717541	1.095254	-3.87443	Li	1.751278	0.682077	-3.830252
Li	3.266651	-0.509354	5.855875	Li	3.209437	-2.249835	3.651987
Li	3.022598	-4.332749	-4.658073	Li	0.474193	-4.441991	-3.563018
Li	-5.541121	3.251514	-1.392548	Li	-3.16538	4.358487	-1.863804
Li	-3.928477	-3.618605	3.828639	Li	-4.522168	-2.971514	2.151678
Li	3.54091	1.163256	1.646994	Li	3.654333	0.359729	1.543145
Li	-1.731477	3.411213	0.24894	Li	-0.792608	3.628555	0.317366
Li	-3.794831	-1.114646	0.945829	Li	-3.819326	-0.217777	1.112323
Li	6.1826	-2.039533	2.275585	Li	5.57292	-2.004218	0.346549
Li	-4.511524	2.964392	3.300476	Li	-3.623718	3.088362	1.852545
Cl	-4.517901	5.089554	0.912084	Cl	-2.989914	6.008804	0.732933
O	-4.892026	6.37975	1.530965	O	-3.141482	7.379623	1.268131
O	-5.008723	5.043476	-0.513437	O	-3.408	5.991107	-0.719272
O	-5.162531	3.940403	1.651491	O	-3.878207	5.063364	1.504189
O	-3.026619	4.9245	0.940973	O	-1.564376	5.556572	0.834593
Cl	5.912582	0.512674	4.14947	Cl	6.161749	-1.049937	3.219031
O	5.231449	-0.320144	5.205378	O	5.215744	-1.992405	3.917366
O	6.805977	1.505224	4.785799	O	7.281806	-0.708065	4.125578
O	4.880907	1.203442	3.31764	O	5.426204	0.184659	2.793798
O	6.73657	-0.412183	3.285017	O	6.7096	-1.72093	1.985918
Cl	-6.338986	-2.070852	2.653547	Cl	-6.712829	-0.824425	2.384866
O	-5.178219	-1.141254	2.381004	O	-5.412527	-0.054209	2.426034
O	-5.845577	-3.164135	3.586349	O	-6.389716	-2.304523	2.452645
O	-6.816333	-2.678534	1.38476	O	-7.438596	-0.532739	1.119039
O	-7.421979	-1.316055	3.334182	O	-7.536297	-0.457477	3.567075
O	-0.202446	4.419462	-3.133391	O	0.696915	4.730949	-3.049323
C	-0.90385	5.59677	-2.635534	C	0.967846	5.895267	-2.225216
C	0.444541	4.698015	-4.407239	C	1.233862	4.920905	-4.38127
C	-0.419419	6.743103	-3.520883	C	2.036721	6.684718	-2.97919
H	-1.977647	5.41241	-2.74207	H	0.037486	6.472786	-2.128441
H	-0.659553	5.701451	-1.57677	H	1.267315	5.521779	-1.243365
C	-0.140962	6.034689	-4.858268	C	1.678916	6.386497	-4.445874
H	1.524175	4.756005	-4.228579	H	2.077131	4.233374	-4.509986
H	0.228178	3.860618	-5.072956	H	0.45155	4.664253	-5.101913
H	0.502859	7.178213	-3.117937	H	3.03521	6.299571	-2.741056
H	-1.166236	7.538306	-3.603848	H	2.013849	7.752656	-2.737237
H	0.548978	6.587565	-5.502714	H	2.517507	6.532687	-5.134684
H	-1.075137	5.876669	-5.409708	H	0.852445	7.029153	-4.774313
O	4.761693	2.280596	-1.865721	O	5.487875	1.329603	-1.796946

C	5.946513	1.635394	-2.403239	C	6.265502	1.98307	-0.753989
C	5.072512	3.649489	-1.461138	C	5.755821	1.951274	-3.077663
C	6.756109	2.793546	-2.973377	C	7.22976	2.925883	-1.479413
H	6.473444	1.126429	-1.586497	H	5.566579	2.510481	-0.099563
H	5.603575	0.899448	-3.130434	H	6.77165	1.203439	-0.174418
C	6.514074	3.901928	-1.930629	C	6.445271	3.277563	-2.754595
H	4.338436	4.295577	-1.950006	H	6.411479	1.286463	-3.65783
H	4.948287	3.71565	-0.378333	H	4.798398	2.059442	-3.59164
H	6.359938	3.085411	-3.953137	H	8.163467	2.409174	-1.735451
H	7.814967	2.543573	-3.089196	H	7.481847	3.801968	-0.872583
H	6.636952	4.907757	-2.34255	H	7.084333	3.62469	-3.57361
H	7.213185	3.79219	-1.09411	H	5.699308	4.05315	-2.544685
O	1.522382	4.503826	2.046982	O	2.598224	4.180723	2.331531
C	2.738297	4.736818	2.819173	C	3.743344	3.95125	3.206188
C	0.747526	5.728429	1.924684	C	1.792604	5.281353	2.833709
C	2.724161	6.23138	3.160239	C	3.729715	5.10509	4.214294
H	2.687557	4.094397	3.701569	H	3.607506	2.975494	3.677608
H	3.59019	4.442588	2.202417	H	4.640626	3.931175	2.583192
C	1.231067	6.596938	3.080531	C	2.240551	5.486966	4.278623
H	0.964316	6.177545	0.947892	H	1.99591	6.162613	2.211259
H	-0.305582	5.45292	1.973461	H	0.751264	4.981636	2.720883
H	3.294605	6.800255	2.417099	H	4.320216	5.951347	3.841808
H	3.159643	6.426533	4.144507	H	4.138964	4.804067	5.184097
H	1.061698	7.661688	2.893932	H	2.074299	6.514222	4.619112
H	0.713632	6.320876	4.006551	H	1.693369	4.811728	4.946718

Table S3. Cartesian coordinates for optimized geometries for $\{THF_2SiFe_3W_9\}$ + 7 Li^+ + N_2.

**$\{THF_2N_2Fe_3W_9\}$ + 3e- / q = -3 / -4683.37444232 a.u.**			
Si	-0.085938	-0.008079	0.100342
W	1.29307	2.265681	-2.350016
W	1.202698	-1.475746	-2.912472
W	-2.319602	2.068719	-1.708405
W	0.18368	3.533097	0.538901
W	-0.043906	-3.565732	-0.595859
W	-2.445015	-1.241018	-2.230569
W	-3.554741	0.087765	0.736811
W	-1.071646	1.193531	3.264285
W	-1.234245	-2.068669	2.709388
O	-0.20083	5.184254	0.958391
O	-3.109774	3.308307	-2.576704
O	-0.566036	1.979736	-2.529261
O	-1.453096	3.0015	-0.246157
O	-2.650138	1.089935	1.963395
O	2.775575	-1.884346	-2.178321
O	-2.89015	0.461066	-2.7802

O	1.525647	0.377965	-2.881868
O	1.660645	3.047997	-3.830973
O	1.45213	-1.82284	-4.571781
O	0.816185	3.782117	-1.257156
O	1.612319	-3.736756	0.046655
O	0.805284	1.356606	-0.156161
O	-0.707186	-3.122376	1.207998
O	-0.59738	-5.18651	-0.69592
O	-1.57977	0.146286	-0.590791
O	-0.750371	-1.107799	-2.980125
O	0.723379	-1.281563	-0.58891
O	-2.160469	-3.259384	3.603356
O	-1.732078	-2.661093	-1.150649
O	0.520607	-3.244471	-2.34486
O	-2.747678	-1.482875	1.512174
O	-3.782306	-1.174915	-0.83192
O	-3.316253	-2.152977	-3.435234
O	-0.262959	-0.280979	1.705548
O	-1.72377	-0.555613	3.761647
O	-3.766628	1.494611	-0.450762
O	-5.139772	-0.023215	1.379588
O	-0.37375	2.589911	2.147215
O	-1.902533	2.136138	4.43348
O	0.36287	-2.212259	3.458265
O	1.89246	3.398118	1.093805
O	0.494613	0.924517	4.039581
O	2.740401	1.091612	2.295036
O	2.67553	-1.963424	1.816042
O	2.908218	2.178071	-1.537435
O	3.623394	-0.104568	-0.233573
Fe	2.00962	-0.585278	3.171317
Fe	2.759099	-1.751048	0.04985
Fe	2.848392	1.449282	0.539653
Li	1.645048	2.416532	2.939259
Li	-0.673206	7.008452	0.594055
Li	-3.021626	-3.334101	5.332325
Li	-4.040643	-1.595652	-5.108985
Li	3.675961	0.275294	-2.057657
Li	1.502114	-3.361463	2.093294
Li	-3.118809	-2.840279	0.200367
N	2.994424	-0.93383	4.728318
N	3.623739	-1.157136	5.66614
O	4.847088	2.4346	0.71249
C	5.637076	2.729932	-0.437214
C	5.720156	1.743397	1.611135
C	6.989596	3.180768	0.129937

H	5.739129	1.815637	-1.038528
H	5.104321	3.478137	-1.021175
C	7.043295	2.512085	1.527211
H	5.233683	1.736983	2.585465
H	5.821114	0.70778	1.261805
H	7.026314	4.270466	0.218677
H	7.815026	2.869968	-0.517288
H	7.102963	3.267045	2.31666
H	7.900063	1.841436	1.640101
O	4.668775	-2.902447	-0.165525
C	5.579549	-2.466742	-1.175833
C	5.415171	-3.014697	1.05655
C	6.840969	-3.273523	-0.897825
H	5.104998	-2.654023	-2.138132
H	5.745355	-1.386997	-1.054287
C	6.864987	-3.341712	0.642907
H	5.340491	-2.067827	1.599222
H	4.932658	-3.790928	1.651252
H	7.735907	-2.804082	-1.317045
H	6.74474	-4.27495	-1.329987
H	7.554204	-2.6009	1.060297
H	7.179665	-4.326029	1.000688

Table S4. Faradaic efficiencies in the three-electrode setup at a function of time, Figure 8a.

	Solvent		
Time, h	PEG-400	Glyme	THF
1	7	7	5
2	7	9	10
3	9	8	7
4	8	6	6
5	8	8	8
